# Predicting responses to platin chemotherapy agents with biochemically-inspired machine learning

**DOI:** 10.1038/s41392-018-0034-5

**Published:** 2019-01-11

**Authors:** Eliseos J. Mucaki, Jonathan Z. L. Zhao, Daniel J. Lizotte, Peter K. Rogan

**Affiliations:** 1 Department of Biochemistry, Schulich School of Medicine and Dentistry, Western University, London, ON N6A 2C1 Canada; 2 Department of Computer Science, Faculty of Science, Western University, London, ON N6A 2C1 Canada; 3 Department of Epidemiology & Biostatistics, Schulich School of Medicine and Dentistry, Western University, London, ON N6A 2C1 Canada; 4 Cytognomix, Inc., London, ON N5X 3X5 Canada; 5 Department of Oncology, Schulich School of Medicine and Dentistry, Western University, London, ON N6A 2C1 Canada

**Keywords:** Predictive medicine, Predictive markers

## Abstract

The selection of effective genes that accurately predict chemotherapy responses might improve cancer outcomes. We compare optimized gene signatures for cisplatin, carboplatin, and oxaliplatin responses in the same cell lines and validate each signature using data from patients with cancer. Supervised support vector machine learning is used to derive gene sets whose expression is related to the cell line GI_50_ values by backwards feature selection with cross-validation. Specific genes and functional pathways distinguishing sensitive from resistant cell lines are identified by contrasting signatures obtained at extreme and median GI_50_ thresholds. Ensembles of gene signatures at different thresholds are combined to reduce the dependence on specific GI_50_ values for predicting drug responses. The most accurate gene signatures for each platin are: cisplatin: *BARD1*, *BCL2*, *BCL2L1*, *CDKN2C*, *FAAP24*, *FEN1*, *MAP3K1*, *MAPK13*, *MAPK3*, *NFKB1*, *NFKB2*, *SLC22A5*, *SLC31A2*, *TLR4*, and *TWIST1*; carboplatin: *AKT1*, *EIF3K*, *ERCC1*, *GNGT1*, *GSR*, *MTHFR*, *NEDD4L*, *NLRP1*, *NRAS*, *RAF1*, *SGK1*, *TIGD1*, *TP53*, *VEGFB*, and *VEGFC;* and oxaliplatin: *BRAF*, *FCGR2A*, *IGF1*, *MSH2*, *NAGK*, *NFE2L2*, *NQO1*, *PANK3*, *SLC47A1*, *SLCO1B1*, and *UGT1A1*. Data from The Cancer Genome Atlas (TCGA) patients with bladder, ovarian, and colorectal cancer were used to test the cisplatin, carboplatin, and oxaliplatin signatures, resulting in 71.0%, 60.2%, and 54.5% accuracies in predicting disease recurrence and 59%, 61%, and 72% accuracies in predicting remission, respectively. One cisplatin signature predicted 100% of recurrence in non-smoking patients with bladder cancer (57% disease-free; *N* = 19), and 79% recurrence in smokers (62% disease-free; *N* = 35). This approach should be adaptable to other studies of chemotherapy responses, regardless of the drug or cancer types.

## Oncology: Computers decode cancer drug responses

Machine learning has identified genetic signatures that predict how patients will respond to three of the most widely used cancer drugs. Chemotherapy regimens are usually based on how groups of people with similar cancers respond to them, but genetic differences can render the drugs more or less effective in individual patients. Machine learning provides a way of sifting through large amounts of data to identify patterns—in this case, in gene signatures associated with cancer recurrence and remission. The authors investigated cellular responses to cisplatin, carboplatin, and oxaliplatin and identified signatures in 11–15 genes which were the most predictive for each drug. The compositions of these signatures are also tailored to how well these therapies prevent growth of cancer cells. Accuracy varied, but one cisplatin signature was able to predict all instances of disease recurrence in non-smokers with bladder cancer.

## Introduction

Chemotherapy regimens are selected based on overall outcomes for specific types and subtypes of cancer pathology, progression to metastasis, other high-risk indications, and prognosis,^1,2^ and variability in tumor resistance has led to the use of tiered, sequential strategies for the selection of agents based on their overall efficacy.^3^ Our group and other researchers have developed machine learning (ML)-based gene signatures (i.e., predictive models) aimed at predicting responses to specific chemotherapeutic agents and minimizing chemoresistance based on the inhibition of growth or drug targets (GI_50_ or IC_50_, respectively).^4–6^ In this study, we present integrated ML models of platin drug responses (cis-, carbo-, and oxaliplatin), and evaluate them using clinical outcome data that were not used to construct the signatures. Previous studies have reviewed the genes,^7^ gene products,^8^ and specific individual pathways that are activated and repressed by drugs,^9^ but comprehensive models of the global cellular response to drugs are lacking. We use integrated ML-based signatures based on the expression of multiple genes to predict key responses to each of these platin agents, for the first time, at different resistance levels.

Cisplatin, carboplatin, and oxaliplatin are each widely prescribed compounds with antineoplastic effects. While each drug contains platinum and forms adducts with tumor DNA, their effectiveness differs for specific types of cancers, such as bladder (cisplatin), ovarian (cisplatin and carboplatin), and colorectal cancer (oxaliplatin). Carboplatin differs in structure from cisplatin, exchanging the dichloride ligands in the latter with a cyclobutane dicarboxylic acid (CBDCA) group, while oxaliplatin is paired with both a diaminocyclohexane (DACH) ligand and a bidentate oxalate group. These chelating ligands have greater stability and solubility in aqueous solutions, which lead to differences in drug toxicity compared to cisplatin.^10^ Oxaliplatin is up to two times more cytotoxic than cisplatin, but it forms fewer DNA adducts.^11^ The large hydrophobic DACH ligand that overlaps with the major groove is thought to prevent the binding of certain DNA repair enzymes, such as the POL polymerases, and may contribute to the low cross-resistance between oxaliplatin and the other two platin drugs.^10^ While all three drugs enter the cell via copper transporters, organic cation transporters are oxaliplatin-specific and likely play a role in its efficacy in colorectal cancer (CRC) cells where these transporters are commonly overexpressed.^7^ Oxaliplatin specifically interferes with both DNA and RNA synthesis, unlike cisplatin, which only infers with DNA.^12^ These intrinsic properties of the platinum drugs lead to differences in their activity and resistance profiles, despite their similar modes of action.

We derived gene signatures to predict drug responses at different sensitivity and resistance levels for each of these agents. Our group and other researchers have used supervised learning algorithms, including random forest models;^13^ support vector machine (SVM) models;^6^ neural networks;^14^ and linear regression models^5^ for these predictions. Pathway and network analyses of gene expression (GE) have been used to identify hundreds of genes that are potentially up- and down-regulated upon cisplatin treatment.^15^ Cisplatin-specific gene signatures have been developed with integrative approaches such as elastic net regression using the inferred pathway activity obtained from data from bladder cancer cell lines.^16^ These methods have implicated genes that have not been described previously. Supervised ML with biochemically relevant genes has also been useful for predicting drug response.^6^ A concern with each of these ML approaches is that an insufficient number of samples coupled with a large number of features, i.e., GE changes, in each sample may result in overfitting of the model, affecting its generalizability with other sources of data.^17^ We therefore reduce the number of dimensions by selecting genes that are biologically relevant to the drugs under observation.^6,17^ In this study, genes included in the final signatures have well-defined roles in responses to their corresponding drugs (Supplementary References, Section A). Additional selection criteria are necessary when the number of genes implicated in peer-reviewed reports is still prohibitively large compared to the sample size.

Biochemically-inspired gene signatures have shown good performance in predicting treatment responses. A paclitaxel ML signature based on tumor GE had a higher success at predicting the pathological complete response rate (pCR^18^) for sensitive patients (84% of patients with no/minimal residual disease) than gene signatures based on a differential GE analysis.^6^ For gemcitabine, a signature derived from both expression and copy number (CN) data from breast cancer cell lines was derived and subsequently applied to the analysis of nucleic acids from archived patient samples. Multiple other outcome measures used to validate gene signatures include prognosis,^5^ Miller–Payne response,^19^ and disease recurrence. Binary SVM classifiers based on discrete time thresholds have been used to classify continuous outcome measures such as prognosis and recurrence. In contrast, pCR is simpler to interpret with binary SVM models. Nevertheless, differences in clinical recurrence have been noted between patients with known pCR and those who do not exhibit disease pathology.^18^ This source of variability in defining patient responses can confound the transferability of SVM models between different datasets.

We apply biochemically-inspired ML to predict and compare the cellular and patient responses to cisplatin, carboplatin, and oxaliplatin. We train models and perform model selection for the classification of platin resistance using data from cancer cell lines, and validate the results using patient GE and clinical outcome data. Our previous gene signatures derived from cell lines were based on median GI_50_ values for each drug.^6^ Models (i.e., gene signatures) learned and selected using the cell line data have not been re-trained prior to application to the patient data since GI_50_ values are not available for patient samples. This approach has been a necessary compromise; however, in the present study, we derive different signatures at the highest and the lowest levels of drug resistance. A series of candidate gene signatures are derived by shifting the GI_50_ thresholds that distinguish sensitivity from resistance. The frequency of genes selected at median and extreme thresholds highlights pathways that most likely define these responses among different patient subsets.

## Results

### Selection of platin drug-related genes

**Fig. 1:**
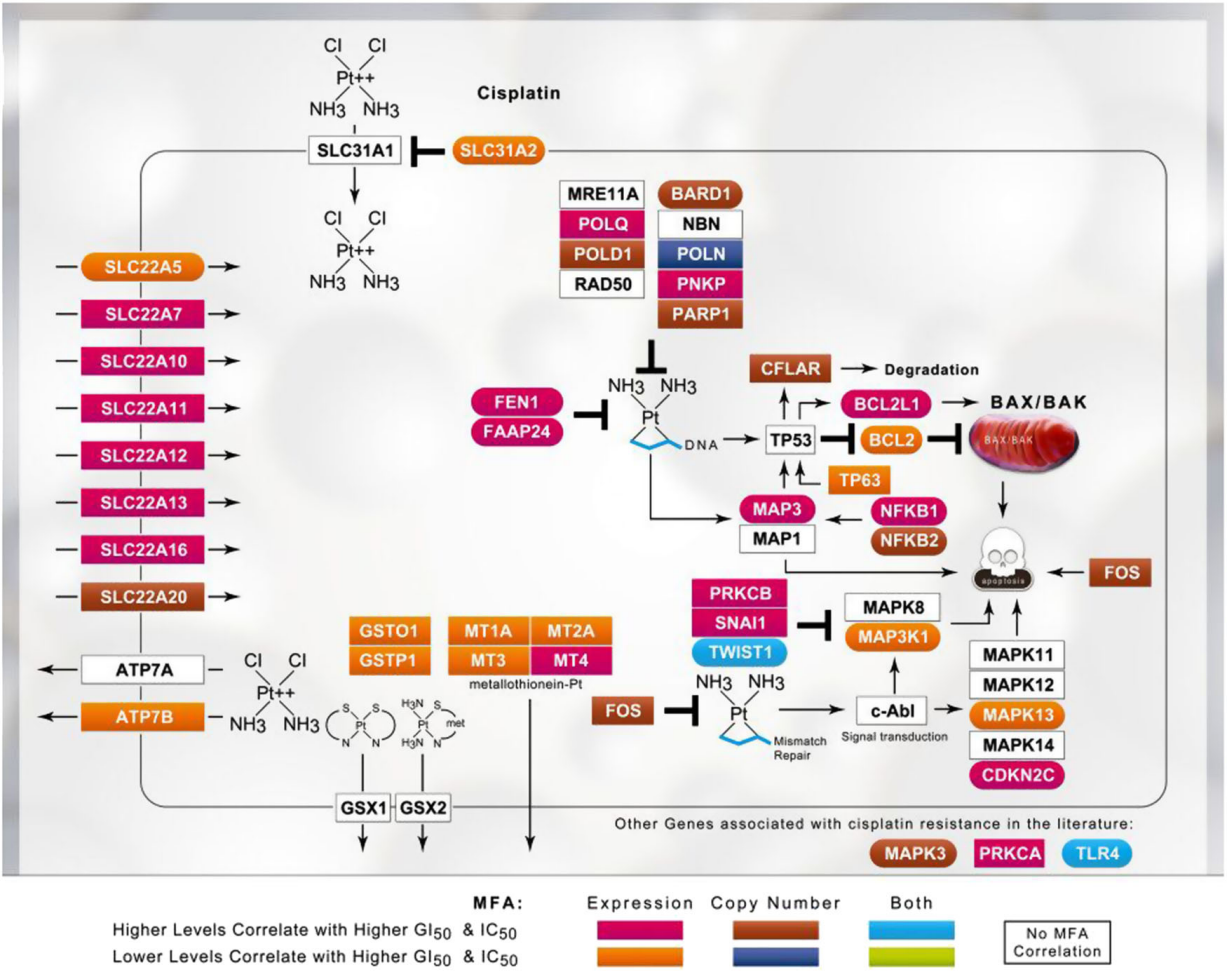
Schematic of platinum drug sensitivity and resistance genes that showed MFA correlation with the GI_50_ values for cisplatin. The gene products corresponding to those used to derive the SVM are indicated within boxes in the context of their cellular mechanisms of action and regulation of drug response. GE and CN correlations with inhibitory drug concentrations are based on the MFA of breast (GI_50_) and bladder (IC_50_) cancer cell line data. Gene products within the best-performing gene signature are embedded within color-coded ovals; whereas the other correlated gene products are embedded within rectangles

**Fig. 2:**
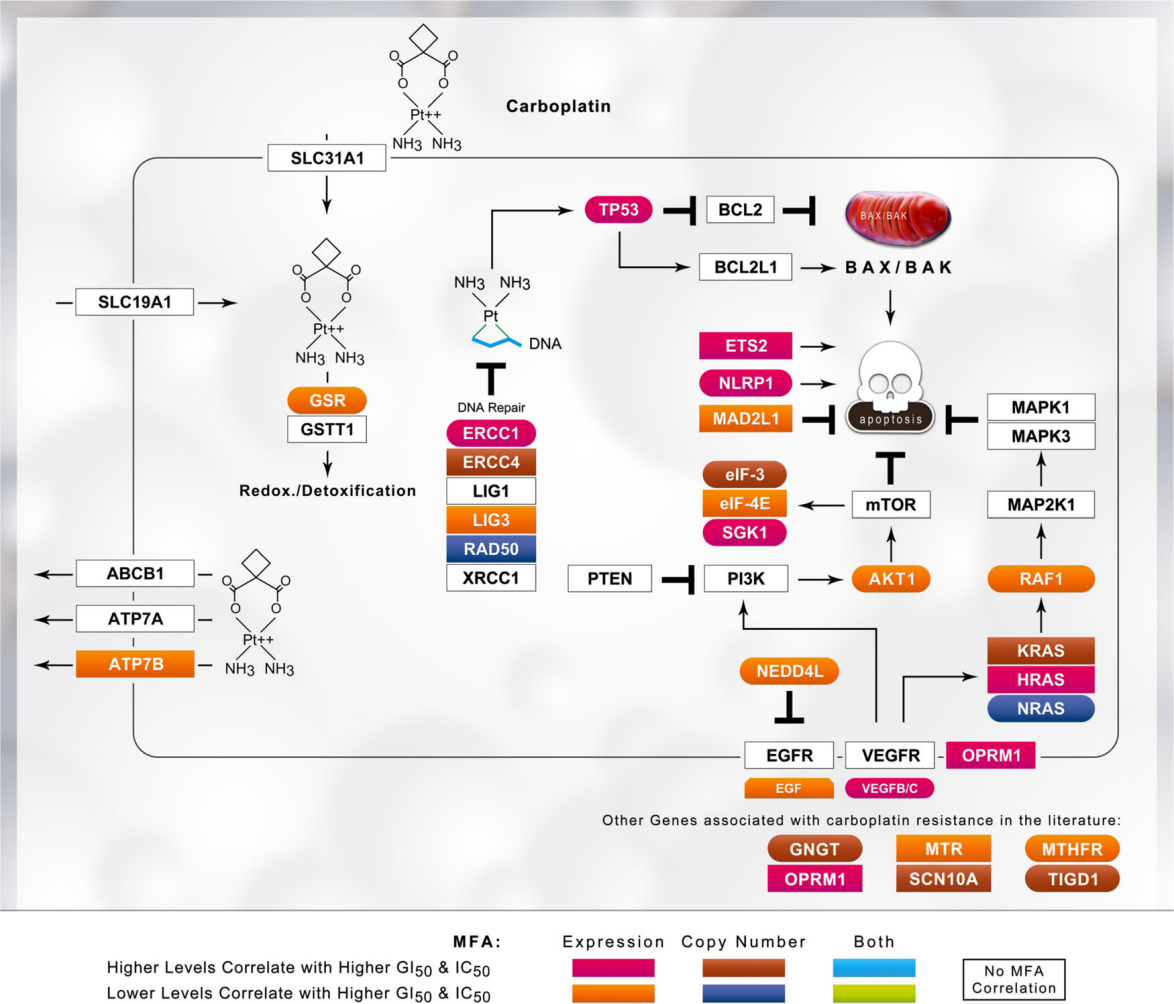
Schematic of platinum drug sensitivity and resistance genes that showed MFA correlation with the GI_50_ values for carboplatin. Refer to the legend of Fig. 1 for details

**Fig. 3:**
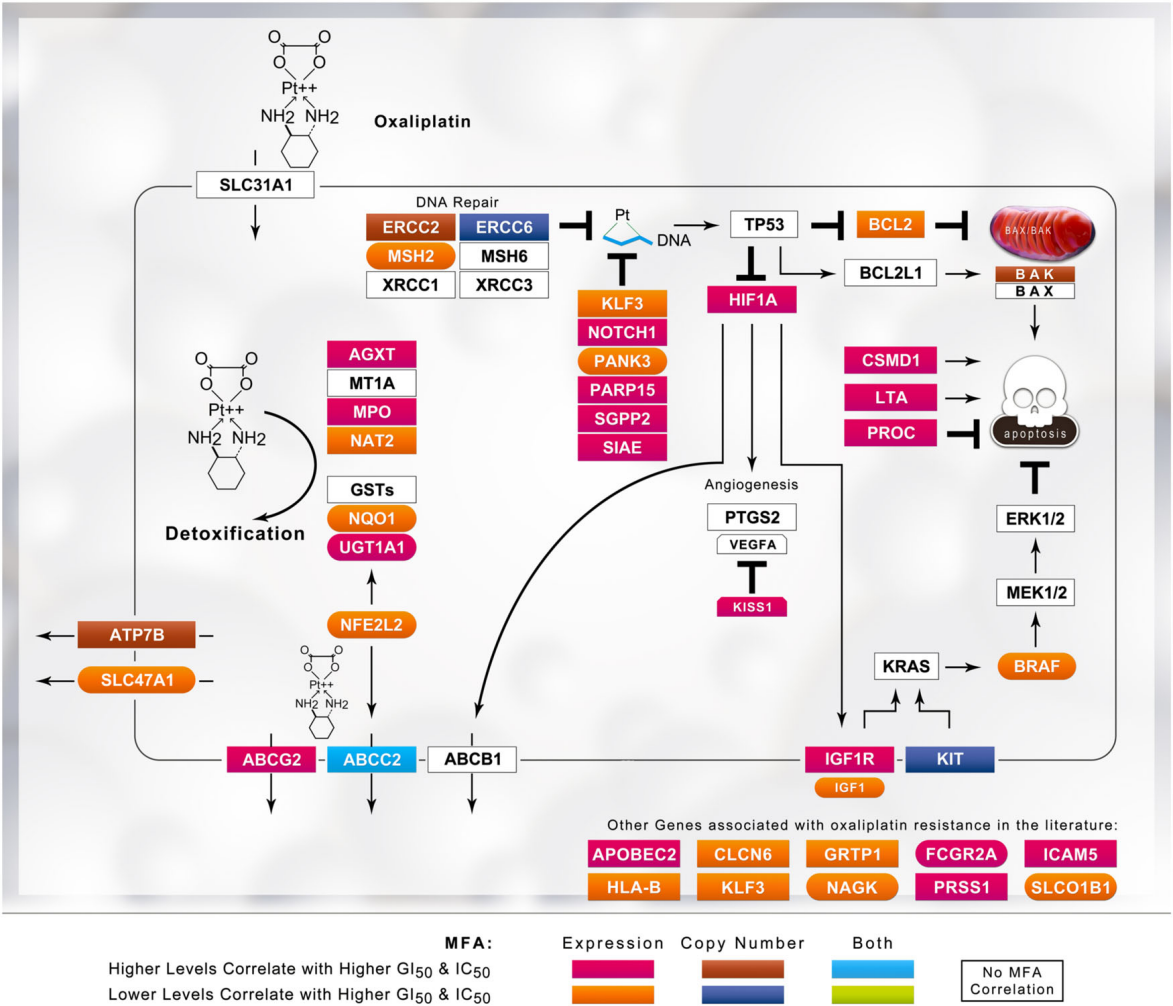
Schematic of platinum drug sensitivity and resistance genes that showed MFA correlation with the GI_50_ values for oxaliplatin. Refer to the legend of Fig. 1 for details

We documented genes in the peer-reviewed literature associated with drug effectiveness or responses (Supplementary References, Section B). For cisplatin, carboplatin, and oxaliplatin, 179, 90, and 288 genes were implicated, respectively (Supplementary Table S1). Multiple factor analysis (MFA) was used to determine which genes correlated with the GI_50_ in breast cancer cell lines through either GE and/or CN,^13^ significantly reducing the sizes of the gene sets for cisplatin (*N* = 39), carboplatin (*N* = 28), and oxaliplatin (*N* = 55). Genes with significant relationships to GI_50_ and the directions of correlations (positive or inverse) are indicated in Figs. 1–3. The diverse functions of these genes included apoptosis, DNA repair, transcription, cell growth, metabolism, immune system, signal transduction, and membrane transport. Analyses of IC_50_ and GE levels for cisplatin-treated bladder cancer cell lines confirmed these relationships based on the GI_50_ values of different breast cancer lines. IC_50_ values were related to GE for *CFLAR*, *FEN1*, *MAPK3*, *MSH2*, *NFKB1*, *PNKP*, *PRKAA2*, and *PRKCA*.^20^ Similarly, IC_50_ values obtained from separate bladder cell lines included in the Genomics of Drug Sensitivity in Cancer project (CancerRxGene; http://www.cancerrxgene.org; *N* = 17)^21^ correlated with GE for *CFLAR*, *FEN1*, and *NFKB1*, as well as *ATP7B*, *BARD1*, *MAP3K1*, *NFKB2*, *SLC31A2*, and *SNAI1*.

**Fig. 4:**
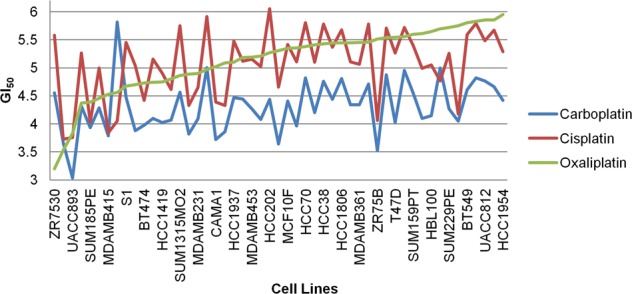
GI_50_ values for cell lines treated with the three platin drugs were plotted in order of ascending oxaliplatin GI_50_. For most cell lines, a trend between the GI_50_ values for cisplatin and carboplatin was observed, reflecting the correlation between the two drugs detected using MFA. Despite this correlation, carboplatin shows a much smaller variance (0.22) than cisplatin (0.37; the oxaliplatin variance is 0.34)

We performed an MFA of the GI_50_ values for cisplatin, carboplatin, and oxaliplatin, without considering either GE or CN. Responses to cis- and carboplatin were directly correlated (a 6.2° separation between vectors), but neither was related to the oxaliplatin response (Fig. 4). Cisplatin-resistant cell lines are generally sensitive to oxaliplatin.^22–24^

SVM-based signatures were initially derived for each platin drug using breast cancer cell line GE data. A 13-gene signature for cisplatin that predicts whether observed growth inhibition is above or below the median GI_50_ threshold (5.2% cross-validation misclassification rate) consisted of *BARD1, BCL2L1, FAAP24, CFLAR, MAP3K1, MAPK3, NFKB1, POLQ, PRKAA2, SLC22A5, SLC31A2, TLR4*, and *TWIST1*. A similarly derived carboplatin signature included *AKT1*, *ATP7B*, *EGF*, *EIF3I*, *ERCC1*, *GNGT1*, *HRAS*, *MTR*, *NRAS*, *OPRM1*, *RAD50*, *RAF1*, *SCN10A*, *SGK1*, *TIGD1*, *TP53*, and *VEGFB* (10.4% cross-validation misclassification). For oxaliplatin, the final SVM gene signature consisted of *AGXT*, *APOBEC2*, *BRAF*, *CLCN6*, *FCGR2A*, *IGF1*, *MPO*, *MSH2*, *NAGK*, *NAT2*, *NFE2L2*, *NOTCH1*, *PANK3*, *PRSS1*, and *UGT1A1* (2.1% cross-validation misclassification). A cisplatin SVM generated from 17 bladder cancer cell lines in CancerRxGene resulted in 2 equally accurate signatures (with 11.8% cross-validation misclassification) consisting of either *PNKP* and *PRKCA*, or *ATP7B*, *CFLAR*, *FEN1*, *MAPK3*, *NFKB1*, and *SLC22A11*. These gene signatures were not useful for predicting patient outcomes due to the limited size of the training set.

### GI_50_ threshold-independent modeling

In our previous studies, we set the median GI_50_ value as the threshold to distinguish drug resistance and sensitivity.^5,6^ An important question is whether the genes contributing to drug responses are consistent among different cell lines, each with their own unique GI_50_ values. Different ML gene signatures were obtained by shifting the GI_50_ threshold, which changed the labels of resistant and sensitive cell lines. After feature selection, the compositions of the corresponding gene signatures for each threshold were compared. Finally, ensemble averaging of all of these optimized SVMs with Gaussian kernels derived for different GI_50_ thresholds was used to create a single aggregated, threshold-independent, ML-based predictive model comprised of all genes that were selected in any of the threshold-specific models (i.e., a composite gene signature).

**Fig. 5:**
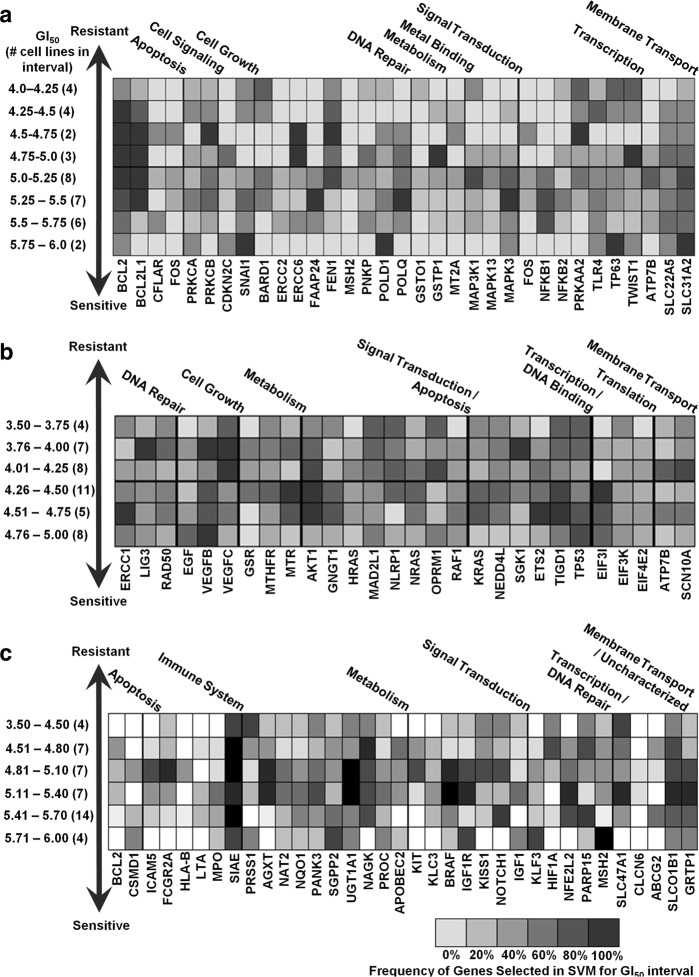
Variation in the composition of the gene signatures obtained using misclassification-based SVMs at different GI_50_ thresholds for **a** cisplatin, **b** carboplatin, and **c** oxaliplatin. GI_50_ intervals are indicated on the left, with the number of cell lines with GI_50_ values within the indicated intervals shown in brackets. Each box represents the density of genes appearing in optimized Gaussian SVM gene signatures in those functional categories, with darker gray indicating frequent genes in the indicated GI_50_ threshold intervals and lighter gray indicating less commonly selected genes. The number of thresholded gene signatures used to derive the density plot within each interval is equal to (or greater than, in the case of multiple equally performing gene signatures) the number of cell lines within that GI_50_ interval

Kinase (*MAPK3* and *MAP3K1*) genes and apoptotic family members (*BCL2* and *BCL2L1*) were the most common genes in the cisplatin signatures at different GI_50_ thresholds, with consistent representation of error-prone and base-excision DNA repair genes as well (Fig. 5a and Supplementary Table S2A). The kinases were more concentrated in signatures with lower drug sensitivity thresholds, whereas *BCL2* and *BCL2L1* were more ubiquitous at all levels. The error-prone polymerases *POLD1* and *POLQ* were more frequently detected in gene signatures with lower sensitivity thresholds, while the flap endonuclease *FEN1* tended to be present at high levels of resistance. Thresholded gene signatures for carboplatin-related genes commonly contained the apoptotic family member *AKT1*, transcription regulation genes *ETS2* and *TP53*, as well as cell growth factors *VEGFB* and *VEGFC*, although the latter were less common at lower sensitivity thresholds (Fig. 5b). Common oxaliplatin-related genes included the transporters *SLCO1B1* and *GRTP1* (but not *SLC47A1*), transcription-related genes *NFE2L2*, *PARP15*, and *CLCN6*, and multiple metabolism-related genes (Fig. 5c).

GI_50_-thresholded ML models were also derived using the log-loss function to evaluate whether an alternative loss function (for classification) would differ significantly from the misclassification-based gene signatures (by both the distribution of selected genes and by model accuracy to patient data). The log-loss function penalizes false classifications, whose value ranges from zero (or completely accurate) to 1 (or completely inaccurate; Supplementary Table S3). The overall distribution of genes across GI_50_ thresholds exhibited many distinct similarities to the gene signatures derived by misclassification. For both sets of cisplatin gene signatures, *BCL2*, *BCL2L1*, and *FEN1* were common in low-to-moderate GI_50_ thresholds, while *NFKB1* was enriched at high thresholds (Fig. 5a and Supplementary Figure S1A). For carboplatin, *AKT1*, *VEGFB*, and *VEGFC* were similarly distributed across GI_50_ thresholds with both methods, although *VEGFB* was less frequently represented in log-loss-based gene signatures at low GI_50_ values (Fig. 5b and Supplementary Figure S1B). In both sets of oxaliplatin gene signatures, *SIAE* and *SLC47A1* were present at high frequencies across all GI_50_ thresholds, whereas *ABCG2* was present less frequently (<50% inclusion; Fig. 5c and Supplementary Figure S1C). Differences between signatures selected by minimizing log-loss and misclassification rates were observed. *EGF* and *ERCC1* were selected at a greater frequency at a moderate carboplatin GI_50_ using the log-loss function, rather than misclassification. Similarly, in oxaliplatin signature genes, *APOBEC2*, *HLA-B*, *LTA*, and *MPO*, were selected considerably more often using the log-loss function. Therefore, while the misclassification- and log-loss-based gene signatures are not interchangeable, overall, they are quite similar to one another.

Log-loss gene signatures were initially constructed either using (a) a modified version of the misclassification-based method, or (b) the backwards feature selection (BFS) software described by Zhao et al.^25^ Multiple signatures with low log-loss values can have different compositions, consistent with the possibility that various diverse gene combinations may give rise to signatures with satisfactory performance. However, these signatures often contain a larger number of gene features than the misclassification-based signatures, raising the possibility that they might be more prone to overfitting. This concern was addressed by generating gene signatures by minimizing log-loss using both methods. The median GI_50_-thresholded cisplatin gene signature generated using the log-loss modified software [*ATP7B*, *BCL2L1*, *CDKN2C*, *CFLAR*, *ERCC2*, *ERCC6*, *FAAP24*, *FOS*, *GSTO1*, *GSTP1*, *MAP3K1*, *MAPK13*, *MAPK3*, *MSH2*, *MT2A*, *PNKP*, *POLD1*, *POLQ*, *PRKAA2*, *PRKCA*, *PRKCB*, *SLC22A5*, *SLC31A2*, *SNAI1*, *TLR4*, and *TP63*] shares 15/19 genes with the signature generated using the BFS software^25^ [*ATP7B*, *BARD1*, *BCL2*, *BCL2L1*, *ERCC2*, *FAAP24*, *FEN1*, *FOS*, *MAP3K1*, *MAPK13*, *MAPK3*, *MSH2*, *MT2A*, *NFKB1*, *PNKP*, *POLQ*, *PRKCB*, *SLC22A5*, and *SNAI1*].

### Impacts of features in gene signatures

Each gene was independently excluded and model accuracy was reassessed within every SVM signature to determine the contributions of individual genes to the overall cross-validation accuracy of a gene signature (Supplementary Table S2A, S2B, and S2C contain cis-, carbo-, and oxaliplatin gene signatures, respectively). The elimination of *ERCC2*, *POLD1*, *BARD1*, *BCL2*, *PRKCA*, and *PRKCB* consistently significantly increased the misclassification error (average > 16% increase) in moderate threshold cisplatin SVMs (GI_50_ thresholds: 5.1–5.5). ERCC2 and POLD1 perform critical functions in nucleotide and base excision repair, respectively. PRKCA and PRKCB are paralogs with significant roles in signal transduction. BARD1 has been shown to reduce the expression of the apoptotic BCL2 gene in the mitochondria,^26^ and has a key role in genomic stability through its association with BRCA1. The genes *NFKB1*, *NFKB2*, *TWIST1*, *TP63*, *PRKAA2*, and *MSH2* showed a high variance in increased misclassification between different gene signatures. The variance of these genes may be due to epistatic interactions with other biological components, including the other genes in the SVM. For example, *NFKB1* and *NFKB2* are jointly included in 7 SVMs generated at a moderate GI_50_ threshold. Possible epistasis was observed, as the removal of either of these genes, but not necessarily both, exerted a substantial impact on model misclassification rates (≥18.0% increase). The misclassification variance of *NFKB1* with *NFKB2* was significantly lower than in SVM gene signatures lacking *NFKB2*.

### Derivation of gene signatures from data obtained from patients with bladder carcinoma

Gene signatures derived from cell line data were validated using data from patients with cancer. We also developed SVMs using the cisplatin and/or carboplatin-treated TCGA (The Cancer Genome Atlas) data from patients with bladder urothelial carcinoma using post-treatment time to relapse as a surrogate criterion for different GI_50_ resistance thresholds to explore the similarities in the gene signatures in the data obtained from these patients (as performed in Mucaki et al.;^27^ Supplementary Table S4). Similar trends to cell line SVMs were apparent: *POLQ* was frequently included in gene signatures with a recurrence threshold of a longer duration, while *FEN1* was a marker of resistance when the time to relapse was shorter. However, *BCL2*, which is present in a majority of breast cancer cell lines SVMs, was present in only one gene signature derived from TCGA data. Similarly, *MSH2* was rarely selected using cell lines, yet appeared in nearly all patient-derived SVMs with >1-year recurrence. However, independently-derived patient SVMs were not able to be used for any other analyses.

### Validation of cell line-based models using data from patients with cancer

GI_50_-thresholded models for each platin drug, which were generated with the breast cancer cell line data, produced 70 cisplatin, 83 carboplatin, and 83 oxaliplatin SVM gene signatures. Each of the thresholded gene signatures was applied to available platin-treated patient datasets to understand how the choice of GI_50_ threshold for training on cell line data impacted the predictive accuracy when the resulting gene signatures were assessed according to patient outcomes.^28–32^ In this study, cisplatin gene signatures were validated using data from patients with bladder cancer, carboplatin signatures were validated using data from patients with ovarian cancer, and oxaliplatin signatures were validated using data from patients with CRC. While the available data contained the necessary GE information, the clinical response metadata differed between studies. The responses of patients with bladder cancer to cisplatin were described as post-treatment survival by Als et al.,^31^ whereas patients with CRC treated with oxaliplatin were categorized as responders and non-responders by Tsuji et al.^32^ TCGA provided two different measures that we used to assess the predictive accuracy of our gene signatures—clinical response to chemotherapy and disease-free survival. Signature accuracy was similar using either measure (Supplementary Table S5A); however, recurrence and disease-free survival were used as the primary measures of responses, as these outcomes were more consistently recorded among the TCGA datasets tested. Patients in the study by Als et al.^31^ with a ≥5-year post-treatment survival were labeled as sensitive to treatment. The differences between these metadata may partially contribute to differences in the prediction accuracy of the thresholded SVM gene signatures.

At higher resistance thresholds for any platin drug (low GI_50_), where more cell lines were labeled sensitive, the positive class (disease-free survival) was correctly classified, while the negative class (recurrence) was highly misclassified (Supplementary Figures S2 and S3). The reverse was true for gene signatures derived using lower resistance thresholds (high GI_50_). For these reasons, SVMs generated at these extreme thresholds were not very useful at predicting patient outcomes. When used to predict recurrence in the TCGA datasets, sensitivity and specificity appeared to be maximized in gene signatures where the GI_50_ threshold for resistance was set near (but not necessarily at) the median (Supplementary Figure S2 and Supplementary Table S5A to S5C). While this pattern held true for the data reported by Tsuji et al.,^32^ oxaliplatin gene signatures, where GI_50_ thresholds were set above the median, better separated patients with primary and metastatic CRC (best signature predicting 92.6% of metastases and 60.7% of primary cancers; Supplementary Table S5C). Although less consistent, cisplatin gene signatures generated with thresholds above the median GI_50_ performed better when evaluating the patient dataset reported by Als et al. (Supplementary Figure S3).^31^

Gene signatures were individually evaluated for their accuracy in TCGA patients using various recurrence times post-treatment to classify resistant and sensitive patients (0.5–5 years; Supplementary Table S6A-C). The best-performing cisplatin signature (hereby identified as **Cis1**; Table 1) accurately predicted 71.0% of the recurrence of bladder cancer in patients who experienced recurrence after 18 months (*N* = 31; 58.5% accurate for disease-free patients [*N* = 41]). The best-performing carboplatin gene signature (designated **Car1** [Table 1]) predicted the recurrence of ovarian cancer after 4 years at an accuracy of 60.2% (*N* = 302; 61.0% accurate for disease-free patients [*N* = 108]). For oxaliplatin, the best-performing gene signature (designated **Oxa1** [Table 1]) accurately predicted 71.6% of the disease-free TCGA patients with CRC after 1 year (*N* = 88; 54.5% accuracy in predicting recurrence [*N* = 11]). These gene signatures (based on GE measured with the Affymetrix Gene Chip Human Exon 1.0 ST arrays), TCGA sample expression data, and SVMs based on bladder cell line data (based on expression measured using the Affymetrix U133A microarray) were added to the online web‐based SVM calculator (http://chemotherapy.cytognomix.com; introduced in Dorman et al.^6^) to predict platin responses.

**Table 1:** Gene signatures that best predicted the responses of TCGA patients

Gene signature ID	Cancer type tested	GI_50_ threshold	Signature (C; σ)
**Cis1** (Cisplatin)	Bladder	5.11	*BARD1, BCL2, BCL2L1, CDKN2C, FAAP24, FEN1, MAP3K1, MAPK13, MAPK3, NFKB1, NFKB2, SLC22A5, SLC31A2, TLR4, TWIST1* (100,000; 100)
Cis2 (Cisplatin)	Bladder	5.12	*BARD1, BCL2L1, CFLAR, FAAP24, MAP3K1, MAPK3, NFKB1, POLQ, PRKAA2, SLC22A5, SLC31A2, TLR4, TWIST1* (10,000; 100)
Cis3 (Cisplatin)	Bladder	5.60	*BCL2, CFLAR, ERCC2, ERCC6, FAAP24, FEN1, MAP3K1, NFKB1, NFKB2, PNKP, POLQ, PRKCB, SLC22A5, SNAI1, TLR4* (100,000; 100)
Cis12 (Cisplatin)	Bladder	5.40	*ATP7B, BCL2, BCL2L1, CDKN2C, ERCC2, FAAP24, GSTO1, MAP3K1, MAPK3, MT2A, NFKB1, NFKB2, POLD1, POLQ, PRKCB, SNAI1, TLR4, TP63* (10,000; 100)
Cis14 (Cisplatin)	Bladder	5.16	*BARD1, BCL2, BCL2L1, CDKN2C, FAAP24, FEN1, FOS, GSTP1, MAP3K1, MAPK13, MAPK3, MSH2, NFKB1, POLD1, POLQ, PRKAA2, PRKCB, SLC22A5, SLC31A2, SNAI1, TWIST1* (10,000; 100)
Cis17 (Cisplatin)	Bladder	5.10	*ATP7B, BCL2, BCL2L1, FEN1, GSTP1, MAP3K1, MAPK3, MT2A, NFKB1, PNKP, POLQ, PRKAA2, PRKCB, SLC31A2, TLR4, TP63* (100,000; 100)
**Car1** (Carboplatin)	Ovarian	4.22	*AKT1*, *EIF3K*, *ERCC1*, *GNGT1*, *GSR*, *MTHFR*, *NEDD4L*, *NLRP1*, *NRAS*, *RAF1*, *SGK1*, *TIGD1*, *TP53*, *VEGFB*, *VEGFC* (100,000; 100)
Car9 (Carboplatin)	Ovarian	4.32	*AKT1, ATP7B, EIF3I, ETS2, GNGT1, HRAS, KRAS, LIG3, MTHFR, MTR, NRAS, RAD50, SCN10A, TIGD1, TP53, VEGFB* (10,000; 100)
Car51 (Carboplatin)	Ovarian	4.34	*AKT1, EGF, EIF3I, ERCC1, ETS2, GNGT1, KRAS, MTHFR, MTR, NEDD4L, NLRP1, NRAS, RAD50, RAF1, SGK1, TIGD1, TP53, VEGFB, VEGFC* (10,000; 100)
Car73 (Carboplatin)	Ovarian	4.09	*AKT1, ATP7B, ETS2, GNGT1, HRAS, NLRP1, SCN10A, VEGFB* (100,000; 1000)
**Oxa1** (Oxaliplatin)	Colorectal	5.10	*BRAF*, *FCGR2A*, *IGF1*, *MSH2*, *NAGK*, *NFE2L2*, *NQO1*, *PANK3*, *SLC47A1*, *SLCO1B1*, *UGT1A1* (10; 10)
Oxa21 (Oxaliplatin)	Colorectal	5.10	*BRAF, IGF1, IGF1R, KLF3, MSH2, NAT2, NFE2L2, NQO1, PANK3, PRSS1, SIAE, SLC47A1, SLCO1B1, UGT1A1* (1000; 100)

C—the box-constraint; *σ*—the kernel-scale (sigma)

Bolded gene signatures are those that exhibited the best overall performance in discriminating among TCGA patient outcomes

The TCGA bladder cancer dataset contained 19 patients treated with carboplatin (but not cisplatin), which enabled an evaluation of the specificity of cisplatin models relative to patients who were not treated with this drug. The cisplatin model that best predicted outcomes of carboplatin-treated patients with bladder cancer in TCGA was not **Cis1** (the best-performing cisplatin model) but rather Cis12 at 2 years post-treatment (80% accurate for responding patients [*N* = 5]; 93% for recurrent patients [*N* = 14]). Cis12 contains 9 genes that are not present in **Cis1**, including *ATP7B*, a gene present in many of our carboplatin models. The presence of this gene may have a significant impact on the overall accuracy of Cis12 in determining the outcomes of the carboplatin-treated patients with bladder cancer. We also evaluated these 19 patients to determine the carboplatin-specific gene signatures, and the signature that best predicted the response of these patients (Car73) was 84% accurate for patients after 1 year of treatment (100% for responding patients [*N* = 11]; 62.5% accuracy for recurrent [*N* = 8]). Interestingly, Car73 shares the same *ATP7B* gene with Cis12. Two additional carboplatin gene signatures were tied for overall accuracy (84%; Car9 and Car51), but more successfully predicted non-responsive patients (87.5%; 82% accuracy for responding patients). *AKT1, ETS2, GNGT1*, and *VEGFB* were shared among these carboplatin gene signatures.

Distances from the hyperplane for all SVMs generated were determined for patients with a short recurrence time to evaluate the consistency of the predicted responses of TCGA patients with bladder cancer who were treated with cisplatin (<6 months, *N* = 10; Supplementary Figure S4). Despite showing similar levels of resistance to treatment, distances differed between patients. While these patients were expected to be indicated as highly cisplatin-resistant (hyperplane distance < 0), two patients (TCGA-XF-A9SU and TCGA-FJ-A871) were predicted to be sensitive by nearly all SVM gene signatures. Similar variations were also observed in patients with either a long recurrence time (>4 years) or no recurrence after 6 years (Supplementary Figure S5).

An aggregate, threshold-independent model was generated for each individual platin drug at different GI_50_ thresholds using ensemble ML, which involves the averaging of hyperplane distances for each model to generate a composite score for each TCGA patient tested (i.e., a composite gene signature). Hyperplane distances across all 70 cisplatin gene signatures were similar, with a mean score of −0.22 and a standard deviation of 3.5 hyperplane units (hu) across the set of patient data. The ensemble model classified disease-free patients diagnosed with bladder cancer with 59% accuracy and those with recurrent disease with 47% accuracy. Limiting ensemble averaging to only cisplatin gene signatures generated at a moderate GI_50_ threshold (ranging from 5.10 to 5.50) did not significantly improve accuracy (44% for disease-free patients and 66% for recurrent patients; Supplementary Table S7A). For carboplatin, ensemble ML did not produce significantly better predictions than random, regardless of the GI_50_ threshold interval selected (Supplementary Table S7B) or the similar mean hyperplane distances (−0.11 ± 3.9 hu). For oxaliplatin, the ensemble ML model (mean = −0.12 ± 2.7 hu) was most accurate after 1 year (60% accuracy for disease-free patients and 73% for recurrent patients; Supplementary Table S7C). Similar to cisplatin, limiting this analysis to oxaliplatin SVM gene signatures with moderate GI_50_ thresholds did not significantly increase accuracy.

### K-fold cross-validation

The misclassification-based cisplatin, carboplatin, and oxaliplatin gene signatures were also evaluated with k-fold cross-validation of TCGA data from patients with bladder, ovarian, and colorectal cancer, respectively. This cross-validation approach was independent of the cell line data; namely, the genes and hyper-parameters of signatures were used, but the GE data used were exclusively derived from patients. Patients were evenly distributed in 5 groups with an equal (or near-equal) ratio of disease-free and recurrent patients. The majority of the cisplatin gene signatures showed an overall accuracy >50%. The cisplatin gene signature that performed best under the k-fold analysis (6-resistance level; *BARD1*, *BCL2*, *BCL2L1*, *PRKAA2*, *PRKCA*, *PRKCB*, and *TWIST1*) showed an overall accuracy of 71.2% (84.4% accurate for sensitive patients and 53.9% accurate for resistant patients). The accuracy of the carboplatin and oxaliplatin gene signatures did not exceed 60%. In general, treating the patient data as a held-out test set yielded higher performance estimates than training and evaluating the models on the patient data using k-fold cross-validation.

### Predicting cisplatin responses in patients based on smoking history

Tobacco smoking is known as the risk factor with the greatest contribution to the development of bladder cancer.^33^ Patients with head and neck cancer who smoke while undergoing cisplatin and radiotherapy treatment have been shown to have a shorter overall survival rate.^34^ We therefore subdivided the patients based on their smoking history and tested the thresholded gene signatures (Supplementary Tables S8 and S9). When testing patients who were lifelong non-smokers, the prediction accuracy of **Cis1** predicted all non-smoking patients who were recurrent after 18 months as cisplatin-resistant (*N* = 5). Prediction accuracy for disease-free patients was 57.1% (*N* = 14). Another gene signature (Cis18; Supplementary Table S8) performed equally well for non-smokers, and these two gene signatures shared the genes *BCL2*, *BCL2L1*, *FAAP24*, *MAP3K1*, *MAPK13*, *MAPK3*, and *SLC31A2*. The threshold-independent analysis predicted the disease-free status equally well, but recurrence was less accurate (66.7%). Notably, non-smokers comprised a small subset of the patients tested (*N* = 19). The threshold-independent prediction of recurrence in patients with a smoking history was 46% accurate (*N* = 13), while disease-free patients were correctly predicted at a rate of 58% (*N* = 19). Recurrence in these patients was best predicted by a gene signature built at the median GI_50_ threshold (Cis2). Accuracy improved for both disease-free (57.7–61.9%) and recurrent patients (76.0–78.6%) when excluding patients who quit smoking more than 15 years before the diagnosis. This SVM included the *CFLAR* and *PRKAA2* genes, which were not present in the two gene signatures that performed well for non-smokers.

We gradually altered the expression of each signature gene until the misclassification was corrected to determine which genes in these gene signatures led to the discordant predictions of patient outcomes. Alterations in the expression of *MAP3K1*, *MAPK3*, *SLC22A5*, and *SLC31A2* corrected discordant predictions of patient outcome. Alterations in *BCL2L1* expression were more likely to correct the discordant predictions of **Cis1** (4 of 5) than Cis2 (2 of 4). If the change exceeded ≥3 times the highest or lowest expression of that gene and the prediction remained unchanged between different patients, then the impact of that gene on the signature was considered to be limited. By these criteria, the expression of *PRKAA2*, *NFKB1*, *NFKB2*, and *TWIST1* was not able to be altered to correct a discordant prediction.

### Cytosine methylation levels of genes in cisplatin models

Tobacco smoking has a significant impact on cytosine methylation levels in the genome.^35^ CpG island methylation is associated with smoking in pack years in a subset of the TCGA patients with bladder urothelial carcinoma.^28^ We suspected that the level of methylation measured in the SVMs that performed best for smoking and non-smoking patients might differ and exert possible concomitant effects on GE. When ranking each gene from **Cis1** by the highest methylation level and GE, 88 of 1080 patient–gene combinations showed the expected inverse correlation between methylation levels and GE (i.e., high methylation and low GE). Methylation and GE levels were more frequently inversely than directly correlated (i.e., high methylation and high GE; *N* = 17). However, the direct correlation was more common in patients with a recent smoking history (70.5%). This pattern was also observed for **Cis2**, which best predicted recurrence in smokers. In cases where methylation and GE were directly correlated, we propose that smoking may alter expression through other effects, e.g., mutagenic effects, rather than solely by epigenetic inactivation through methylation.

## Discussion

Using GE signatures, we derived both GI_50_ threshold-dependent and -independent ML models that predict the chemotherapy responses to cisplatin, carboplatin, and oxaliplatin, respectively. The cisplatin gene signature **Cis1** (Supplementary Table S6A) most accurately predicted the response of patients with bladder cancer after 18 months, and **Car1** (Supplementary Table S6B) best predicted the response of patients with ovarian cancer after 4 years. **Oxa1** (Supplementary Table S6C) more accurately predicted disease-free patients than patients with recurrent disease at the 1-year treatment threshold. The thresholds that best represented the time-to-recurrence differed between the platin drugs in patients with each cancer type. Cisplatin gene signatures exhibited noticeably improved performance when smoking history was taken into account.

The three platin drugs produced distinctly different gene signatures. The composition of the initial gene sets exhibited some overlap between platin drugs (*N* = 67 between any two platins), but the expression of only *ATP7B*, *BCL2*, and *MSH2* was correlated with the GI_50_ values of more than one platin drug. The expression of *BCL2L1, GSTP1, MAP3K1, MAPK3, MT1A*, and *MT2* was correlated with cisplatin GI_50_ values, but not with carboplatin and/or oxaliplatin GI_50_. Similarly, the carboplatin GI_50_ was correlated with *AKT1, EGF, ERCC1, KRAS, LIG3, MTHFR, MTR, RAD50*, and *TP53*, while oxaliplatin GI_50_ was correlated with *ATM, BCL2, CLCN6, ERCC2, ERCC6*, and *UGT1A1*. Despite the close similarity between cisplatin and carboplatin GI_50_ responses (see Fig. 4), MFA only related the expression of one gene (*ATP7B*) to GI_50_ levels of both drugs. *BCL2* and *MSH2* correlated with both the cisplatin and oxaliplatin GI_50_ values (*BCL2* levels did not correlate with carboplatin GI_50_). The increase in misclassification caused by the elimination of *MSH2* from any gene signature in which it was present was significant; for example, misclassification of Cis14 and Oxa21 (Table 1) was increased by 28.2% and 19.1%, respectively (Supplementary Table S2A and S2C). These differences may reflect the spectrum of activity, sensitivity, and toxicity of these signature genes.^22–24,36,37^

Our previous validation using patient expression and CN data for other chemotherapy drugs on other datasets^6,27^ exhibited better performance than what is reported in this study. We investigated the possibility that disease and molecular heterogeneity in platin-treated patients may have affected the accuracy of our results. Model predictions were re-evaluated after stratifying clinical features, such as time-to-disease recurrence, cancer stage, and metastatic lymph node count. Patients with advanced stage breast cancer (stage III and IV) were analyzed separately from patients with earlier stage diagnoses (stage I and II). The cisplatin gene signature **Cis1** performed best on stage IV patients (overall accuracy of 72.4% at a 2-year recurrence threshold), while **Oxa1** similarly performed best in predicting late stage cancers (74.5% accurate for stage III and 71.4% accurate for stage IV at a 2-year recurrence threshold). Cis5 was also more accurate for patients with later stage cancers (72.4% overall accuracy at 18 months). The accuracies of gene signatures were similar across all stages (e.g., **Car1** ranged from 58 to 74%). Cisplatin-treated patients with bladder cancer and oxaliplatin-treated patients with CRC in TCGA were also stratified by lymph node status (N0, N1, and N2 [the dataset of patients with bladder cancer only included two N3 patients, which were combined with N2 patients in the analysis; N3 was not presented in patients with CRC]). In TCGA patients with bladder cancer, **Cis1** exhibited ~60% accuracy across all categories; however, it performed better in sensitive N0 and N1 patients than N2. Cis2 was less accurate for N2 patients than for N0 and N1 patients. Sensitive N2 patients were more likely to be misclassified (<40%) than relapsed N2 patients. In TCGA patients with CRC, **Oxa1** was 88% accurate in identifying N2 patients (95% accurate for sensitive N2 patients [*n* = 19], and 67% accurate for relapsed N2 patients [*n* = 6]). Oxaliplatin gene signatures were less accurate for N1 patients than N0 and N2 patients. Thus, heterogeneity in the disease stage and metastatic phenotypes adversely confounds the overall accuracies of our predictions.

Gene signature models derived from cell lines and tested on patients differed in their respective outcome measures. The exact GI_50_ cell line threshold that best predicts patient outcomes is not known, and different groups use different methods to establish thresholds for GI_50_ values.^38,39^ Therefore, we developed ML models for platin drugs that predict drug responses without relying on arbitrarily set GI_50_ thresholds. For cisplatin, SVM ensemble averaging generated at different resistance thresholds showed a small increase in accuracy compared with most gene signatures, better representing the sensitive, disease-free class (59% accuracy). Interestingly, ensemble averaging of only the gene signatures built using a moderate GI_50_ threshold yielded results that better represented the resistance class. This result more closely matched the accuracy of **Cis1**, and may be due to the greater overall impact of **Cis1** on the ensemble prediction. When limiting ensemble averaging to only those gene signatures with the highest area under the curve (AUC) at each resistance threshold, differences in predictions were negligible. Ensemble ML potentially avoids problems with poor performance and overfitting by combining gene signatures that individually perform slightly better than chance.^40^

Reconciliation of gene signatures without features known to be related to chemoresistance with tumor biology is challenging. Our thresholding approach may reveal potentially important genes and pathways associated with platin resistance. A preferable method would be to explore pathways related to signature genes to improve accuracy, identify potential targets for further study of chemoresistance, and expand the model parameters by considering alternate states other than those captured in the original signature.^41^ Signatures for resistance may be useful for developing targeted interventions to re-sensitize tumors. For example, the mismatch repair (MMR) gene *MSH2* is commonly present in gene signatures at high resistance levels for oxaliplatin, which is of interest because MMR deficiency has been shown to predict oxaliplatin resistance.^37^ Indeed, *MLH1-*, *MSH2-*, and *MSH6*-deficient cells are more susceptible to oxaliplatin, although an MMR deficiency is associated with cisplatin resistance.^36^ The autoimmune disease-associated gene *SIAE*, which exhibits a strong negative correlation with the oxaliplatin response in patients with advanced CRC,^42^ was selected in the majority of thresholded oxaliplatin gene signatures (Supplementary Table S2C). The gene *BCL2*, which was commonly selected for cisplatin (Fig. 5a), was rarely selected for oxaliplatin (Fig. 5c). At the highest levels of resistance to cisplatin, gene signatures were enriched for genes belonging to DNA repair, anti-oxidant response, and apoptotic pathways, as well as drug transporters (Fig. 5a). These gene pathways are known to be involved in cisplatin resistance^43,44^ and these specific genes may be explored in subsequent studies designed to identify their contributions to the chemotherapy response in a biochemical context.

Log-loss evaluates the accuracy of a classifier by penalizing erroneous classifications and is relevant in cases where data are imbalanced and/or have an unequally distributed error cost. We assessed whether ML gene signatures based on log-loss minimization improved the accuracy of predicting patient responses (Supplementary Table S3) and compared them to gene signatures generated by minimizing cell line misclassification. When gene signatures generated from both methods were highly similar (generated at the same GI_50_ threshold, consisting of a similar number of genes and ≥80% shared genes), the prediction accuracy of outcomes of TCGA patients with cancer was nearly indistinguishable, as accuracy can vary over different relapse thresholds. When significant differences in predictions were observed, the misclassification-based gene signatures were generally more accurate (**Cis1**, Cis17, and the “12-Resistant” carboplatin gene signature were +8.3%, +5.6%, and +3.9% more accurate than the log-loss gene signature, respectively). Oxaliplatin gene signatures were dissimilar across all GI_50_ thresholds, as the log-loss minimized ML gene signatures often contained greater numbers of genes than the misclassification-based gene signatures. Many of these larger gene signatures were less accurate in predicting patient outcomes than gene signatures that minimized misclassification rates, consistent with the observation that this evaluation and model selection method is more prone to overfitting. This pattern was also noted for gene signatures generated at extreme GI_50_ thresholds for all three platin drugs, in which the response was, by definition, somewhat imbalanced.

The prediction of responses to combination chemotherapy with the gene signatures described here may be feasible. Not included in the present analysis were signatures for methotrexate, vinblastine, and doxorubicin, which comprise the MVAC cocktail used to treat bladder cancer. This lack of analysis was primarily due to the small number of patients treated with this drug combination in the TCGA bladder cancer dataset (*N* = 11). Individual signatures for several of these drugs have been derived and analyzed using the patient data from Molecular Taxonomy of Breast Cancer International Consortium (METABRIC).^27^ A reasonable approach to predicting responses to combination chemotherapy would be to first determine the probability of sensitivity or resistance to individual drugs, after accounting for the misclassification rate for each (defined as d_1_, …, d_k_). The ML classifiers output these probabilities, analogous to their misclassification rates in a set of patients treated identically. If the model predicts that the patient is sensitive to drug d_1_ with 90% probability, sensitive to drug d_2_ with 5% probability, and the errors are independent, then the probability of sensitivity to the combination is 1 − (1 − 0.9) * (1 − 0.05) = 90.5%, and the probability of resistance is 9.5%, assuming no synergistic effects between drugs. If interactions or dependence among errors are suspected, the combined probability of resistance to the pair d_12_ could be estimated based on the features that are shared by the signatures of both drugs. The probability of sensitivity to the combination would then be: 1 − (1 − d_12_) * (1 − d_3_) *…* (1 − d_k_).

The predictive accuracy for the same gene signature might be able to provide good differentiation between the two datasets. Cis3 (Supplementary Table S6A) had an AUC of 0.64 when validated against TCGA patients with bladder cancer. However, the AUC was lower when applied to the dataset reported by Als et al.^31^ (AUC = 0.18). Patient metadata in the latter study only indicated patient survival times, while we based the expected TCGA patient outcomes on time to disease recurrence. As the basis of our expected outcomes differed between datasets, these differences may serve as a confounding factor when determining the accuracy of gene signatures. The datasets also differed in how expression was measured (microarray vs. RNA-seq). The relevance of gene signatures based on training and testing data from different platforms can affect the accuracy of validation, which might not be improved by data normalization. In the present study, datasets were subjected to *z*-score normalization. Other techniques to correct for some of these effects have been described and could be applied in subsequent studies.^45^

In summary, we describe GI_50_ or IC_50_ threshold-independent ML gene signatures that predict the chemotherapy responses of patients with cancer to platin agents. Ensemble ML produced combined signatures that were more accurate than most individual gene signatures generated with different thresholds. Genes associated with cisplatin response included genes that exacerbate resistance in patients with a history of smoking. The methodology described here should be adaptable to other drugs and cancer types. With a range of gene signatures for multiple drugs, the efficacy of treatment might be improved by tailoring treatment to a patient’s specific tumor biology and reduce treatment duration by limiting the number of different therapeutic regimens prescribed before achieving a successful response.^46^

## Materials and methods

### Data and preprocessing

#### Cell-line datasets

Microarray GE and data obtained from breast cancer cell lines were used to train ML-based gene signatures of drug responses based on respective growth or target inhibition data (GI_50_ or IC_50_). Cell lines were treated with either cisplatin (*N* = 39), carboplatin (*N* = 46), or oxaliplatin (*N* = 47).^13^ Bladder cancer cell line GE and IC_50_ measurements for cisplatin were obtained from CancerRxGene (*N* = 17).^46^ However, all models (gene signatures) used to evaluate patient data were trained on breast cancer cell line data because the number of bladder cancer cell lines was insufficient to produce accurate signatures.

#### Datasets from patients with cancer

RNA-seq GE and survival measurements were downloaded from TCGA for patients with bladder urothelial carcinoma (*N* = 72 patients treated with cisplatin),^28^ ovarian epithelial tumors (*N* = 410 treated with carboplatin),^29^ and colorectal adenocarcinoma (*N* = 99 treated with oxaliplatin).^30^ GE values for cisplatin-treated patients with cell carcinoma of the urothelium (*N* = 30)^31^ and oxaliplatin-treated patients with CRC (*N* = 83)^32^ were obtained from the Gene Expression Omnibus. Clinical metadata and GE for TCGA patients were obtained from Genomic Data Commons (https://gdc.cancer.gov/), while methylation HM450 (Illumina) data for these patients were downloaded from cBioPortal.^47^

#### Development and pre-processing of biochemically-inspired gene sets

Initial gene sets used to develop signatures for each drug were identified from previous publications (see Supplementary References, Section B) and databases, such as PharmGKB and DrugBank.^48,49^ The evidence supporting each gene contained in the final signatures is independent scientific evidence that the genes selected are not the result of spurious associations. The final gene sets were chosen using MFA with the breast cancer cell line data to analyze interactions between GE, CN, and GI_50_ data for the drug of interest.^50^ Genes whose GE and/or CN showed a direct or inverse correlation with GI_50_ were selected for SVM training. Because the number of genes related to the GI_50_ for oxaliplatin exceeded the number of cell lines available for training, we limited the input for the oxaliplatin ML model to those genes whose GE were related to the GI_50_. Similarly, the number of correlated genes in cisplatin-treated cells exceeded the number of cell lines. For cisplatin, genes whose expression correlated with the GI_50_ were eliminated if they showed no or little expression in TCGA patients with bladder cancer (i.e., RNA-seq counts by Expectation Maximization [RSEM] were <5.0 for the majority of individuals). This approach reduced the overall number of genes for the SVM analysis, and thus helped to avoid a data to size sample imbalance. For cisplatin, the MFA was repeated using IC_50_ values for 17 bladder cancer cell lines; however, the available CN data for these genes generally showed a lack of variation in the cell lines. Instead, the available IC_50_ values for three other cancer drugs (doxorubicin, methotrexate, and vinblastine) were compared with the IC_50_ of cisplatin using MFA.

The direct application of an SVM model to patient data without a normalization approach is imprecise when training and testing data are not obtained using similar methodology (i.e., different microarray platforms). Expression levels were normalized by conversion to *z*-scores using MATLAB to compare the cell line GE microarray data and the patient RNA-seq GE datasets.^51^ Although log_2_ intensity values from microarray data were not available for TCGA samples, RNA-seq-based GE data and log_2_ intensities from microarray data are highly correlated.^52^

### Machine learning

SVMs were trained with breast cancer cell line GE datasets^13^ with the Statistics Toolbox in MATLAB^51^ using a method similar to the procedure reported by Dorman et al.^6^ Rather than employing a linear kernel, we used a Gaussian kernel function (fitcsvm) and then tested the data with leave-one-out cross-validation (using the options “*crossval*” and “*leaveout*”). A greedy BFS algorithm was used to improve the classification accuracy.^53^ BFS leaves out individual genes from the initial MFA-qualified gene set and then trains a cross-validated Gaussian kernel SVM on the training samples, removing the gene with the highest misclassification rate. The procedure is repeated until all genes have been evaluated. The gene subset with the lowest misclassification rate^6^ or log-loss statistic^25^ based on cross-validation is selected as the gene signature for subsequent testing with patient GE and clinical data. K-fold cross-validation of the misclassification-based gene signatures was performed using MATLAB software, as described in Zhao et al.^25^

SVMs minimized using the log-loss classification function were also generated with both the software described in Zhao et al. (uses a multiclass compatible “fitcecoc” function)^25^ and with a modified version of the software described above (using “fitSVMPosterior” to compute posterior probabilities). Computed probabilities differed between “fitSVMPosterior” and “fitcecoc” (range: 0.02–0.04); thus, the resulting gene signatures will differ between the two programs. When given unbalanced data (e.g., lower resistance thresholds), “fitSVMPosterior” will warn that some classes are not represented, and thus those folds will not predict the labels for those missing classes. The log-loss gene signatures described in this manuscript were generated with the multiclass compatible “fitcecoc” function software.^25^

### Derivation of gene signatures for different drug resistance thresholds

We have previously set a conventional GI_50_ threshold that distinguishes sensitivity from resistance at the *median* of the range of drug concentrations that inhibited cell growth by 50%.^6^ We hypothesized that different gene signatures would be derived for different levels of drug resistance by varying this threshold. ML experiments for classifying resistance or sensitivity at GI_50_ values generated a series of optimized Gaussian SVM gene signatures whose performances were assessed with patient expression data for each signature. A heat map illustrating the frequencies of genes appearing in these gene signatures was created with the R language *hist2d* function.

A composite gene signature was created by ensemble averaging of all gene signatures generated at each resistance threshold. Ensemble averaging combines signatures by averaging the weighted accuracy of a set of related models.^40^ The decision function for the ensemble classifier is the mean of the decision function scores of the component classifiers, weighted by the AUC.

### Significance of cell line-derived gene signatures

The significance of the derived SVMs (regardless of whether the observed performance of the gene signatures could have arisen by chance) was first assessed using a permutation analysis with randomized cell line labels and with random sets of genes, as described previously.^6^ Using the median cisplatin GI_50_ as the resistance threshold, 10,000 gene signatures based on random gene selection (15 genes) had higher rates of misclassification than the best median SVM gene signatures (2 signatures with 7.7% misclassification). Cisplatin, carboplatin, and oxaliplatin GE data for random cell line label combinations (*n* = 10,000) generated only 8, 1, and 1 signatures, respectively, with lower error rates than the best biochemically-inspired signatures. When minimizing for log-loss (rather than misclassification), the random gene analysis (10,000 iterations; median cisplatin GI_50_ threshold) only resulted in gene signatures with a higher log-loss than the signature generated with the initial cisplatin gene set. The log-loss-based random label analysis (*n* = 2000 combinations) resulted in 3.4% of random label gene signatures with a lower log-loss than the cisplatin signature at the same GI_50_ threshold (5.27). This finding was not entirely surprising since it depends on the GI_50_ threshold used for labeling. The differences between GI_50_ values for cell lines close to the median GI_50_ used in this analysis were almost negligible (e.g., 5.11 vs. 5.12) and likely within the measurement error for these values.

Regarding the specificity of the cisplatin gene signatures, the best-performing cisplatin gene signatures (**Cis1** and **Cis2**) were used to evaluate participants who were treated with other drugs (using an 18-month post-treatment threshold). Among these patients, 36.5% of patients who were disease-free were predicted accurately with the **Cis1** signature (*N* = 178; 22% less accurate than platin-treated patients), and 62.9% of patients with recurrent disease were predicted accurately (*N* = 70; 8.1% less accurate). **Cis2** was 43.8% accurate at predicting disease-free non-platin-treated patients (*N* = 178; 12.3% lower accuracy) and 60.0% accurate at predicting patients who relapsed (*N* = 70; 2.9% less accurate). GE changes in patients treated with platin drugs were better modeled by cancer cell-line-based predictors than in patients receiving other drug treatments.

## Supplementary information

Supplementary materials

Supplementary Tables
