# Supplementary material for: Predicting responses to platin chemotherapy agents with biochemically-inspired machine learning
Source: Signal Transduct Target Ther. 2019 Jan 11;4:1. doi: 10.1038/s41392-018-0034-5 (PMC6329797; doi:10.1038/s41392-018-0034-5)
Supplement: Supplementary file 1 — Supplementary materials [file 41392_2018_34_MOESM1_ESM.docx]

**Supplementary materials**

**Predicting Responses to Platin Chemotherapy Agents with Biochemically-inspired Machine Learning**

Eliseos J. Mucaki^1^, Jonathan Z.L. Zhao^1,2^, Daniel J. Lizotte^2,3^, and **^§^**Peter K. Rogan^1,2,3,4,5^

**Author Affiliations**

^1^Department of Biochemistry, Schulich School of Medicine and Dentistry, Western University, London, Canada, N6A 2C1

^2^Department of Computer Science, Faculty of Science, Western University, London, Canada, N6A 2C1

^3^Department of Epidemiology & Biostatistics, Faculty of Science, Western University, London, Canada, N6A 2C1

^4^Cytognomix, Inc., London, Canada N5X 3X5

^5^Department of Oncology, Schulich School of Medicine and Dentistry, Western University, London, Canada, N6A 2C1

Author Emails: [emucaki@uwo.ca](mailto:emucaki@uwo.ca), [jzhao293@uwo.ca](mailto:jzhao293@uwo.ca), [dlizotte@uwo.ca](mailto:dlizotte@uwo.ca), and [progan@uwo.ca](mailto:progan@uwo.ca)

**^§^Correspondence to:** Peter K. Rogan (progan@uwo.ca), Department of Biochemistry, Schulich School of Medicine and Dentistry, Western University, London, Ontario, Canada, N6A 2C1. 1 (519) 661-4255.

**Supplementary Tables.** Details of gene signatures, validation, and accuracy are indicated in Tables: S1A) Genes Selected for MFA of Gene Expression/Copy Number to Cisplatin GI_50_; S1B) Genes Selected for MFA of Gene Expression/Copy Number to Carboplatin GI_50_; S1C) Genes Selected for MFA of Gene Expression/Copy Number to Oxaliplatin GI_50_; S2A) SVM Models Based on Varying Resistance Thresholds and Impacts on Misclassification, Categorized by Gene Function for Cisplatin; S2B) SVM Models Based on Varying Resistance Thresholds and Impacts on Misclassification, Categorized by Gene Function for Carboplatin; S2C) SVM Models Based on Varying Resistance Thresholds and Impacts on Misclassification, Categorized by Gene Function for Oxaliplatin; S3A) Cisplatin SVM Models Derived for a Range of Response Thresholds Using Log-Loss Minimization; S3B) Carboplatin SVM Models Derived for a Range of Response Thresholds Using Log-Loss Minimization; S3C) Oxaliplatin SVM Models Derived for a Range of Response Thresholds Using Log-Loss Minimization; S4) SVM Models Generated with Bladder Cancer Patient Data at Various Time to Recurrence Thresholds, Categorized by Gene Function for Cisplatin; S5A) Cisplatin SVM Models Derived from a Range of Response Thresholds for TCGA Patients with Bladder Cancer Using Misclassification; S5B) Carboplatin SVM Models Derived from a Range of Response Thresholds for TCGA Patients with Ovarian Epithelial Tumors using Misclassification; S5C) Oxaliplatin SVM Models Derived from a Range of Response Thresholds for TCGA Patients with Colorectal Adenocarcinoma Using Misclassification; S6A) Accuracy of SVMs for TCGA Patients with Bladder Cancer in Relation to Time to Recurrence After Treatment With Cisplatin; S6B) Accuracy of SVMs for TCGA Patients with Bladder Cancer in Relation to Time to Recurrence After Treatment With Carboplatin; S6C) Accuracy of SVMs for TCGA Patients with Bladder Cancer in Relation to Time to Recurrence After Treatment With Oxaliplatin; S7A) Accuracy of the Threshold-independent Analysis (Ensemble Averaging) for Cisplatin; S7B) Accuracy of the Threshold-independent Analysis (Ensemble Averaging) for Carboplatin; S7C) Accuracy of the Threshold-independent Analysis (Ensemble Averaging) for Oxaliplatin; S8) Accuracy of SVMs for Non-Smoking Patients with Bladder Cancer in TCGA; S9) Accuracy of SVMs for Smoking Patients with Bladder Cancer in TCGA (within 15 years of diagnosis).

All Supplementary Tables can be found in the accompanying Excel document.

**Supplementary Figures**

**
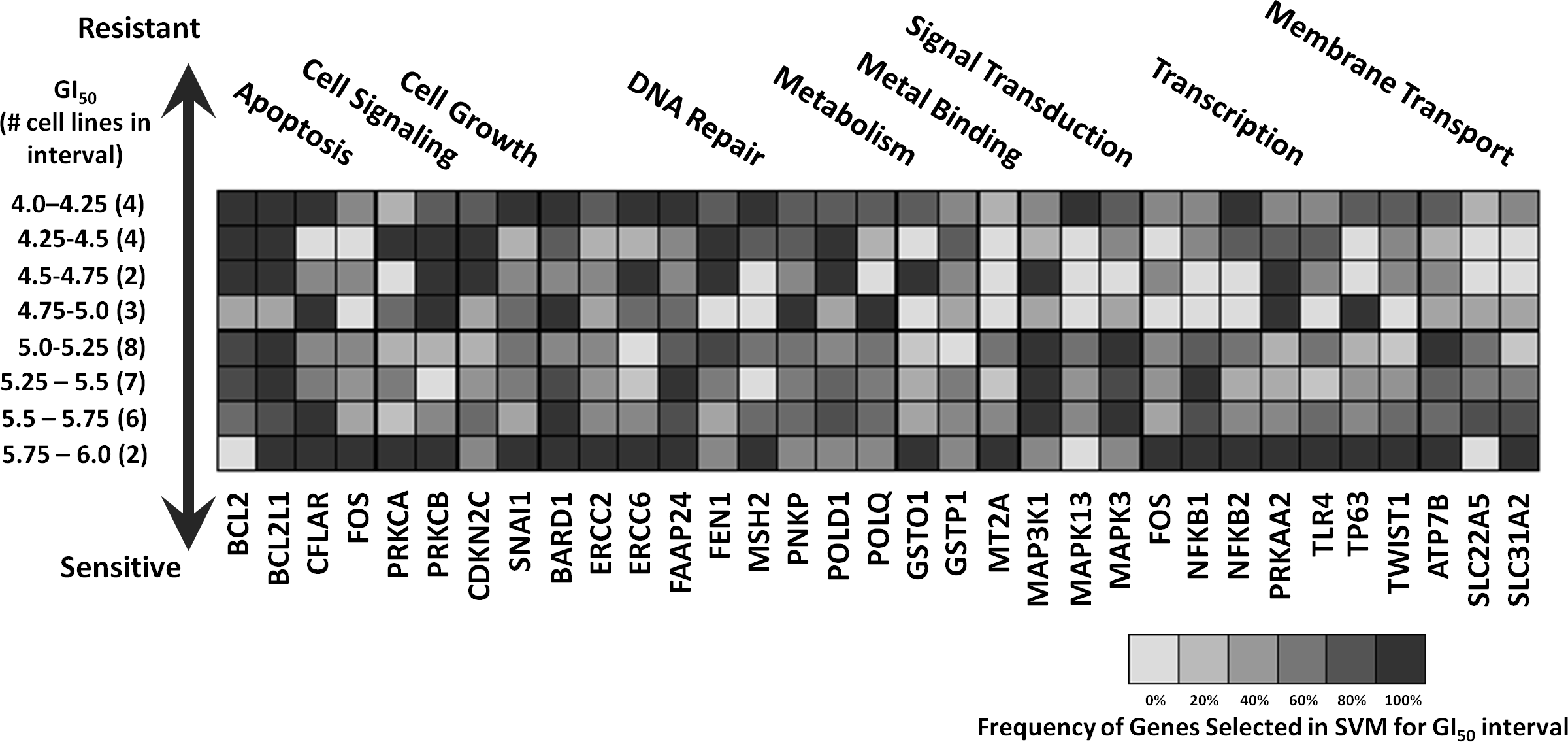
**

**Supplementary Figure S1A.** Variation in the gene composition of log-loss-based SVMs at different GI_50_ thresholds for cisplatin. Each box represents the density of genes appearing in optimized Gaussian log-loss SVM gene signatures in those functional categories, with darker grey indicating frequently detected genes in the indicated GI_50_ threshold intervals and lighter grey indicating less commonly selected genes.

**
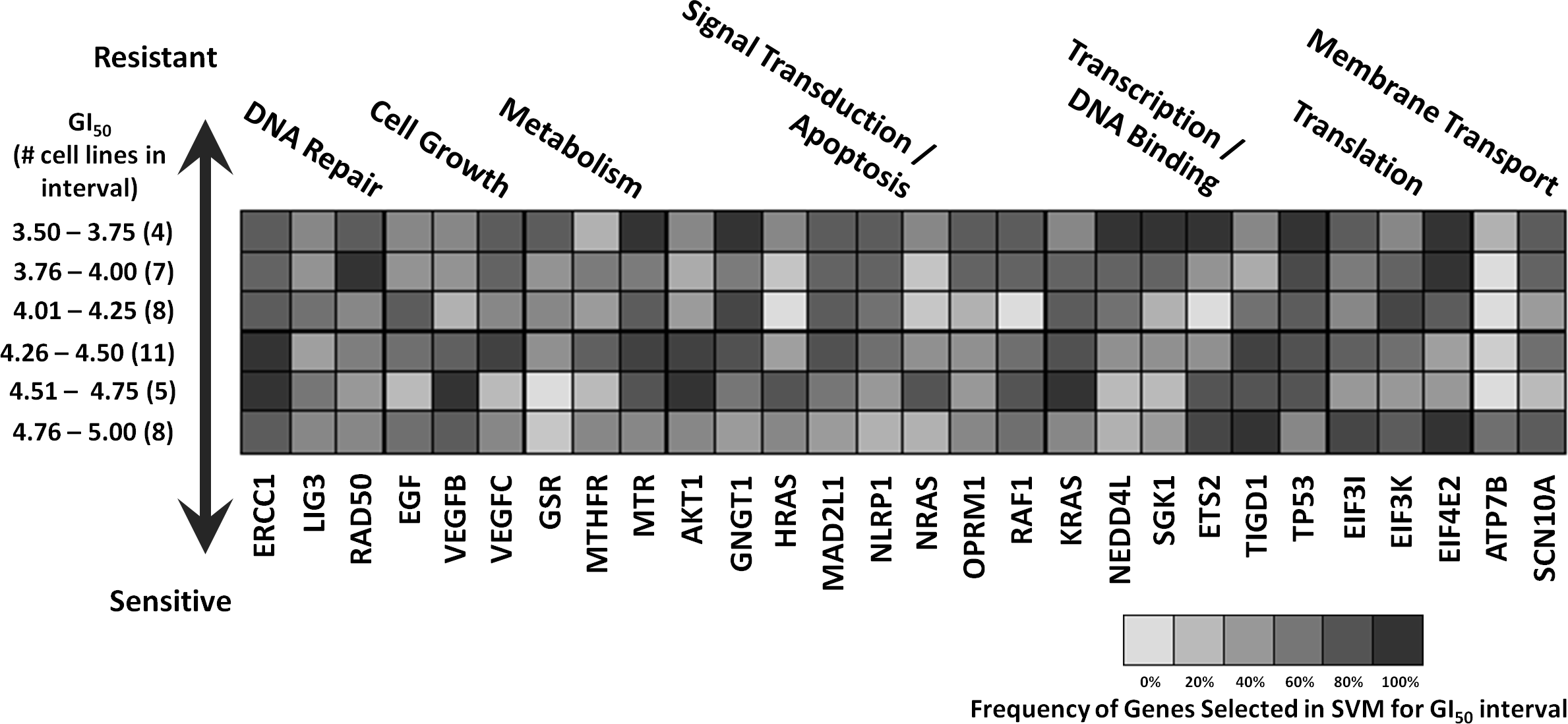
**

**Supplementary Figure S1B.** Variation in the gene composition of log-loss-based SVMs at different GI_50_ thresholds for carboplatin. Each box represents the density of genes appearing in optimized Gaussian log-loss SVM gene signatures in those functional categories, with darker grey indicating frequently detected genes in the indicated GI_50_ threshold intervals and lighter grey indicating less commonly selected genes.

**
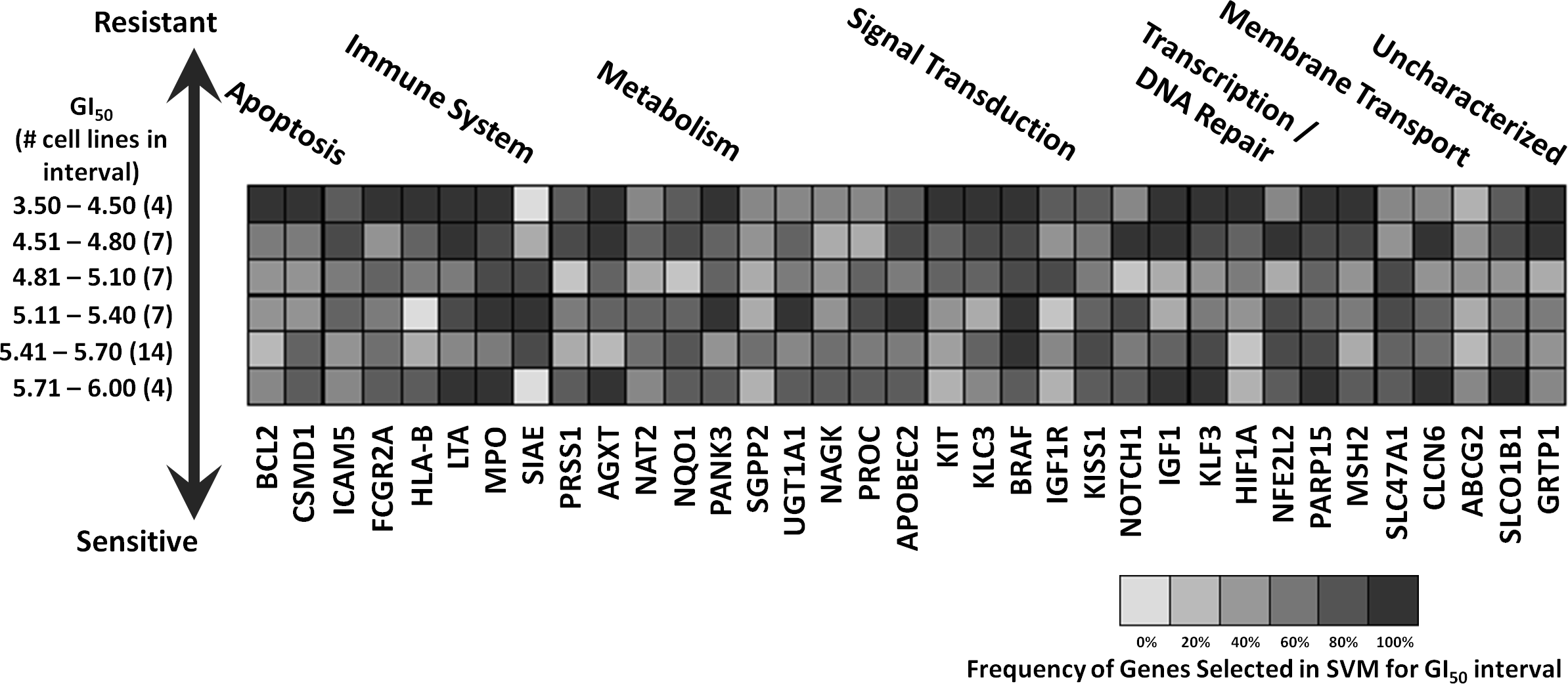
**

**Supplementary Figure S1C.** Variation in the gene composition of log-loss-based SVMs at different GI_50_ thresholds for oxaliplatin. Each box represents the density of genes appearing in optimized Gaussian log-loss SVM gene signatures in those functional categories, with darker grey indicating frequently detected genes in the indicated GI_50_ threshold intervals and lighter grey indicating less commonly selected genes.

**
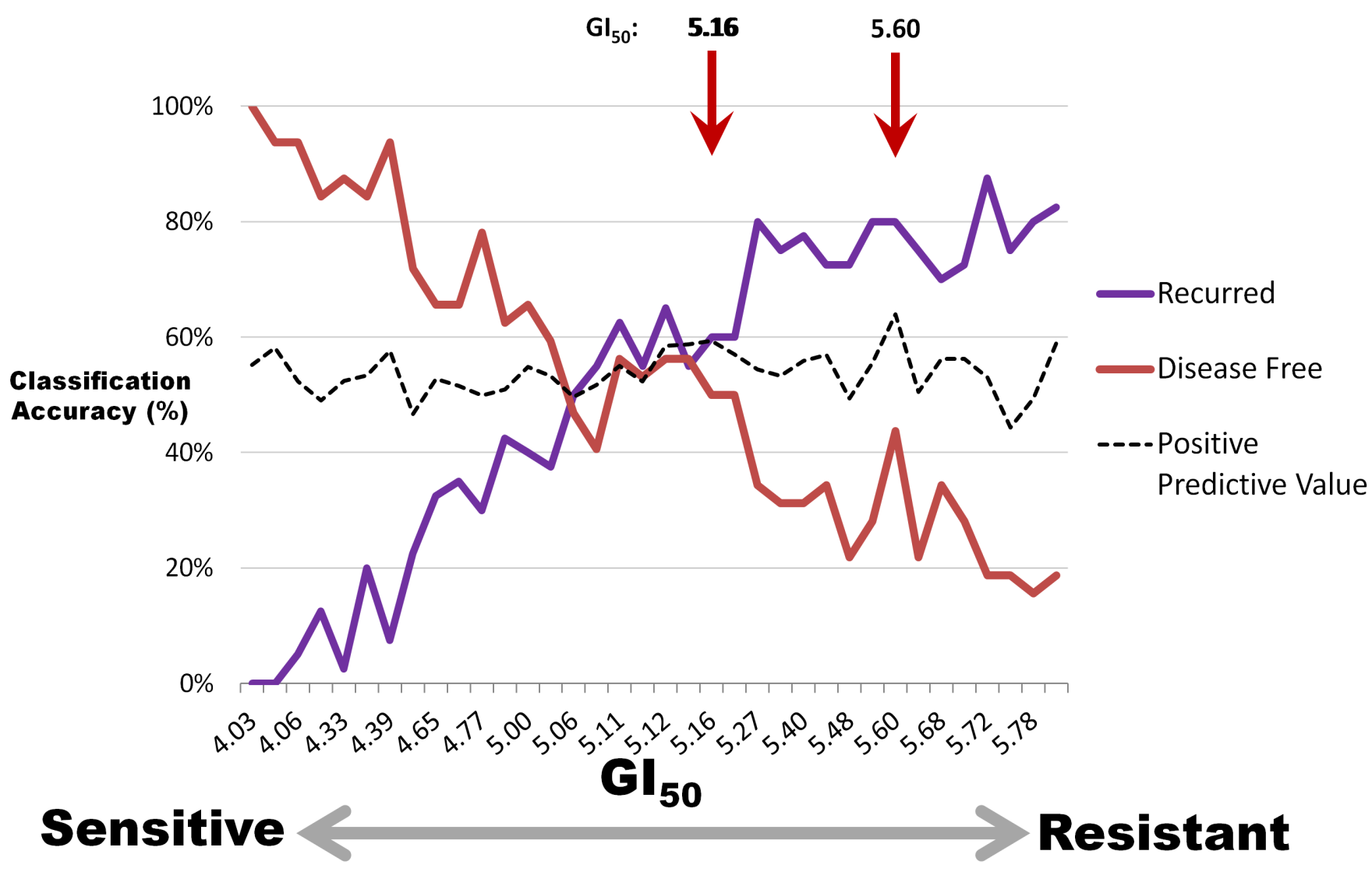
**

**Supplementary Figure S2.** Classification accuracy of gene signatures for TCGA patients with bladder cancer treated with cisplatin and/or carboplatin as the resistance threshold is varied. Recurrence and disease-free survival are used as a binary measure to assess performance. The x-axis indicates movement of the resistance threshold, with more cell lines labeled sensitive on the left and more labeled resistant on the right. The maximal AUC is indicated by the downward arrows.

**
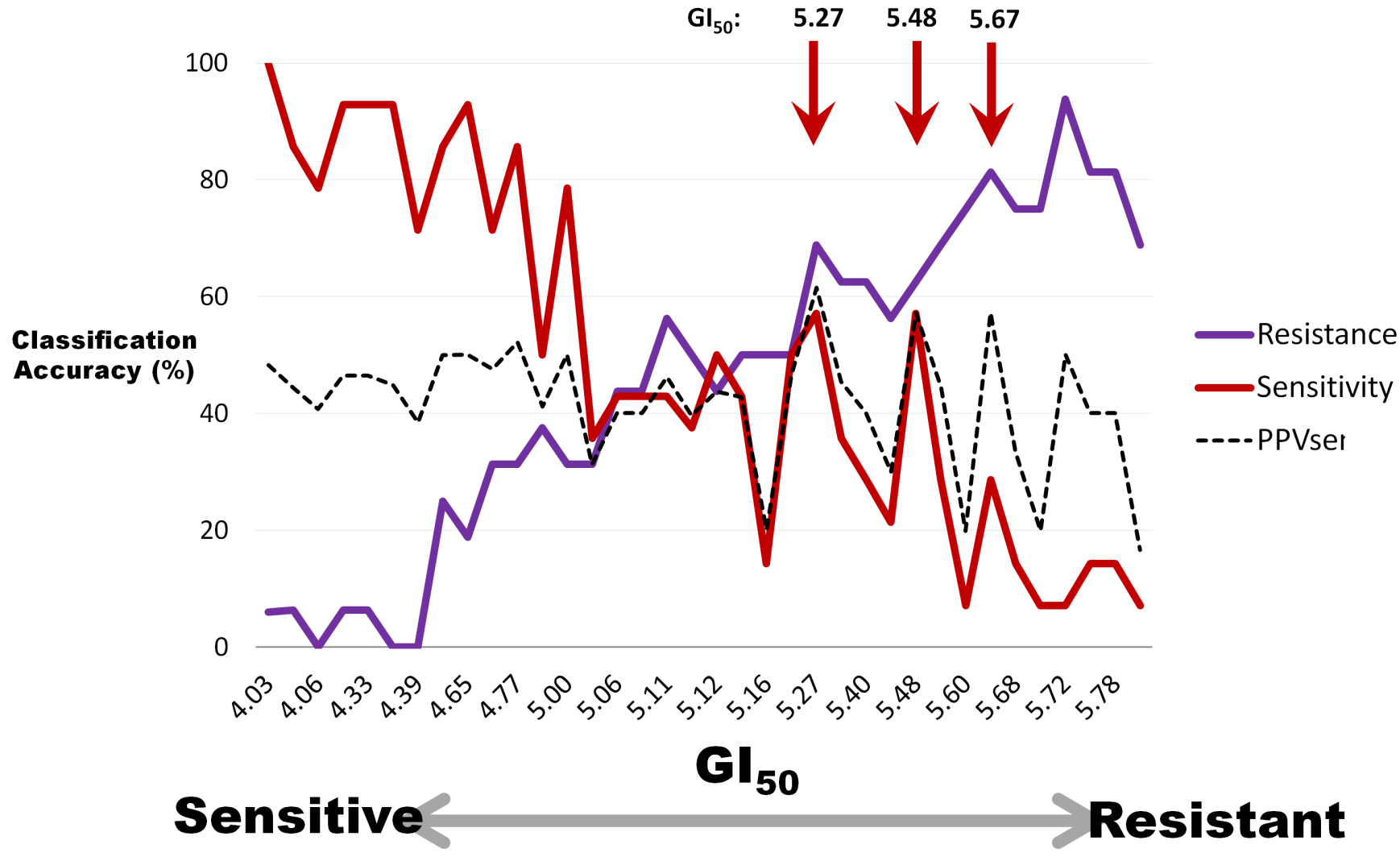
**

**Supplementary Figure S3.** Classification accuracy of SVM gene signatures for cisplatin were assessed at a range of response thresholds using gene expression data for cisplatin-treated patients with bladder cancer reported in the study by Als *et al.* ^29^. Patients with a ≥ 5 year survival post-treatment were labeled sensitive. Red arrows indicate the SVM gene signatures with the highest positive predictive value (PPV) in the accuracy of classifying patient outcomes.

**
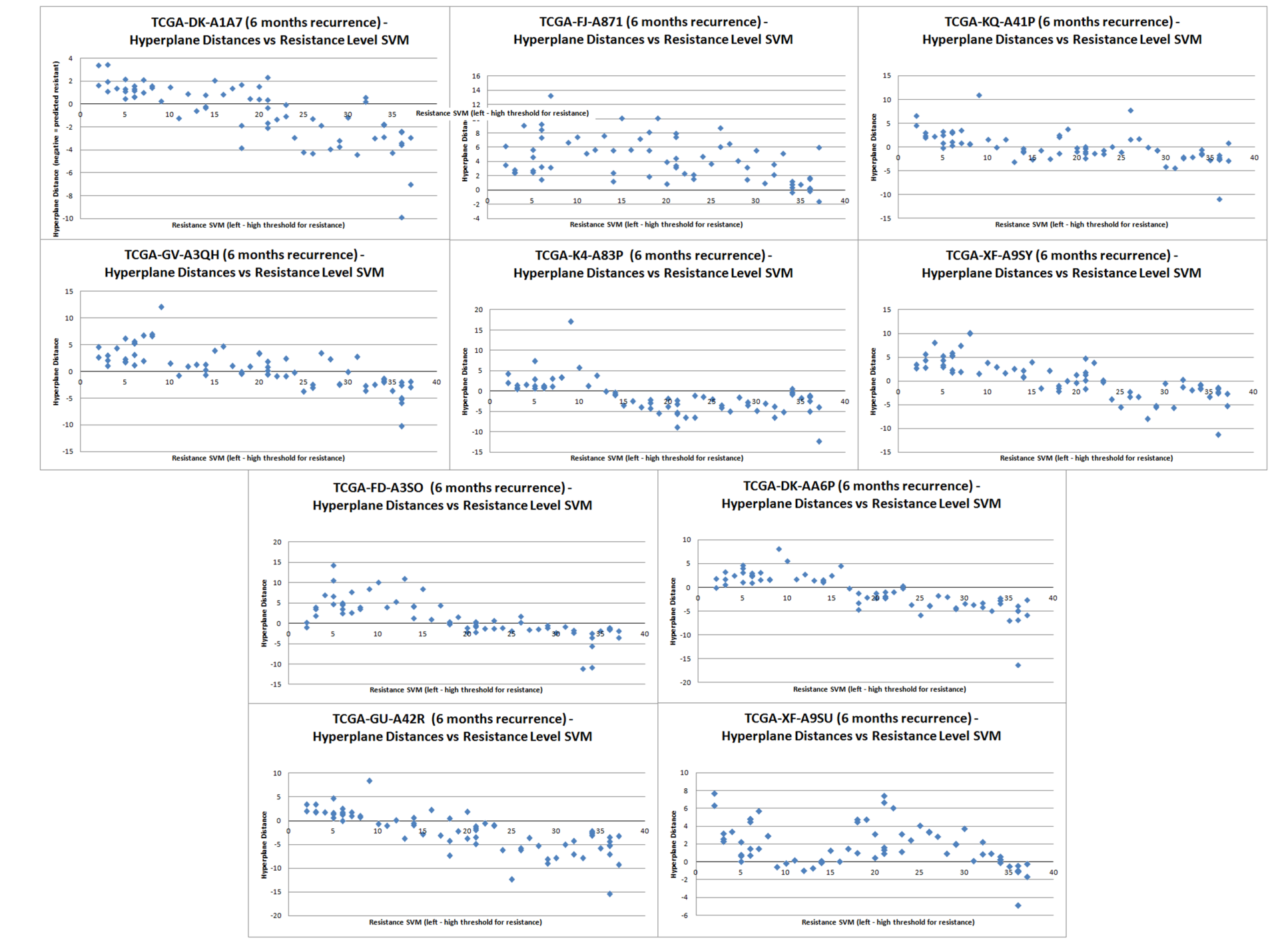
**

**Supplementary Figure S4.** Hyperplane distance calculated by all thresholded SVMs for recurrent (<6 months) patients in TCGA. Each diagram represents the predictions of all SVMs for all patients who experienced recurrence less than 6 months after treatment (N=10). Each point represents an SVM, where the x-axis represents the number of cell lines set to resistant (in order of lowest to highest GI_50_) and the y-axis represents the calculated hyperplane distance. A negative hyperplane distance would represent a prediction of resistance to cisplatin. Nevertheless, some patients showed a strong preference towards predictions of sensitivity (i.e., TCGA-XF-A9SU).

**
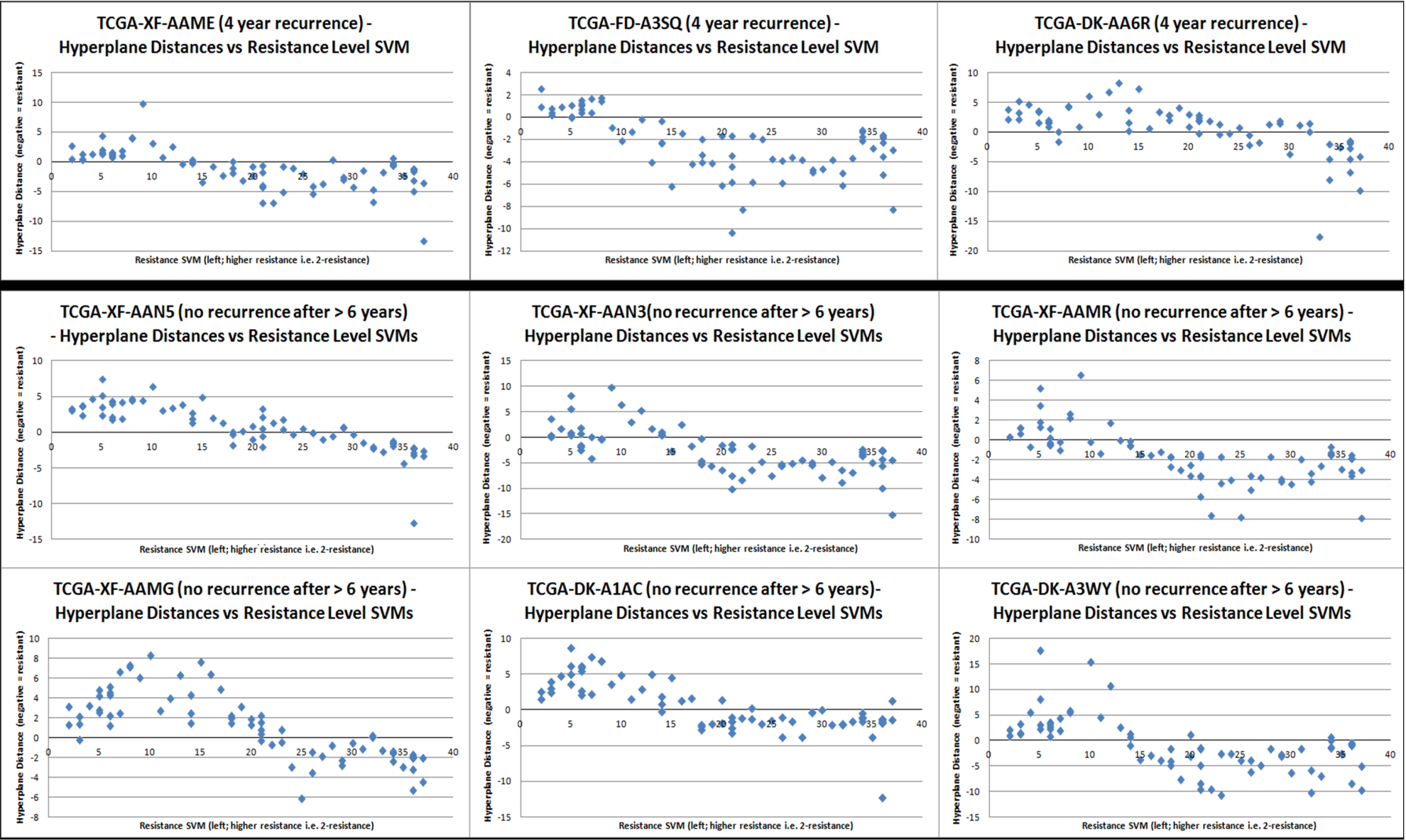
**

**Supplementary Figure S5.** Hyperplane distance calculated by all thresholded SVMs for sensitive patients in TCGA. Each diagram represents the predictions of all SVMs for all patients who experienced recurrence > 4 years after treatment (top panel; N=3) or patients who showed no recurrence after 6 years (bottom panel; N=6). Each point represents an SVM, where the x-axis represents the number of cell lines set to resistant (in order of lowest to highest GI_50_) and the y-axis represents the calculated hyperplane distance. A positive hyperplane distance would represent a prediction of sensitivity to cisplatin.

**Supplementary References.** A) Experimental evidence from studies correlating expression to platinum drug efficiency supporting the inclusion of genes. A subset of genes with a consistent significant increase in misclassification error is shown. B) The initial peer-reviewed literature used to develop gene signatures associated with cis-, carbo- and oxaliplatin responses.

**Supplementary References, Section A.** Biological Justification for Inclusion of Gene in Final Gene Signature

*BCL2 (Cisplatin)*

1. Leisching G, Loos B, Botha M, Engelbrecht AM. Bcl-2 confers survival in cisplatin treated cervical cancer cells: circumventing cisplatin dose-dependent toxicity and resistance. *J Transl Med.* **13:**328 (2015).

- This study observed that cervical cancer cells treated with cisplatin upregulate *BCL2*. When *BCL2* was silenced, cisplatin sensitivity was enhanced, and apoptosis induction was significantly increased.

2. Cho HJ, et al. Upregulation of Bcl-2 is associated with cisplatin-resistance via inhibition of Bax translocation in human bladder cancer cells. *Cancer Lett.* **237(1):**56-66 (2006).

- This study reported that overexpression of *BCL2* inhibits cisplatin-induced Bax translocation. They performed RNAi experiments to down-regulate *BCL2*, which re-sensitized cells to cisplatin.

3. Michaud WA, et al. Bcl-2 blocks cisplatin-induced apoptosis and predicts poor outcome following chemoradiation treatment in advanced oropharyngeal squamous cell carcinoma. *Clin Cancer Res.* **15(5):**1645-54 (2009).

- In this report, high *BCL2* expression was observed to be associated with resistance to cisplatin in HNSCC cell lines. The addition of a *BCL2* expression vector to cell line would also increase cisplatin resistance.

4. Xie Q et al. ABT737 reverses cisplatin resistance by regulating ER-mitochondria Ca2+ signal transduction in human ovarian cancer cells. *Int J Oncol.* **49(6):**2507-2519 (2016).

- In Xie *et al., BCL2* siRNA and ABT737 (a *BCL2* / *BCL2L1* inhibitor) was shown to increase induction of calcium ions by cisplatin in cytosol and mitochondria, consequently inducing apoptosis. Furthermore, ER-mitochondria contact sites were increased in cisplatin-resistant SKOV3/DDP ovarian cancer cells when treated to cisplatin and ABT737 (or the *BCL2* siRNA).

*BARD1 (Cisplatin)*

5. Atipairin A, Ratanaphan A. In Vitro Enhanced Sensitivity to Cisplatin in D67Y BRCA1 RING Domain Protein. *Breast Cancer (Auckl).* **5**:201-8 (2011).

- The *BRCA1*-*BARD1* RING complex plays essential role in E3 ubiquitin ligase function and in response to DNA damage repair. This study showed that a D67Y mutation in *BRCA1* disrupts the E3 ligase activity of the *BRCA1*-*BARD1* RING complex, and cells with this mutation were more sensitive to cisplatin treatment.

6. Atipairin A, Canyuk B, Ratanaphan A. The RING heterodimer BRCA1-BARD1 is a ubiquitin ligase inactivated by the platinum-based anticancer drugs. *Breast Cancer Res Treat.* **126(1):**203-9 (2011).

- This report showed that when BARD1 is platinated by cisplatin in vitro, its E3 ligase activity was not observed (Figure 5; lanes 4 and 5).

7. Coté D, et al. Germline single nucleotide polymorphisms in ERBB3 and BARD1 genes result in a worse relapse free survival response for HER2-positive breast cancer patients treated with adjuvant based docetaxel, carboplatin and trastuzumab (TCH). *PLoS One.* **13(8)**:e0200996 (2018).

- This article reports the *BARD1* SNP rs2229571 is associated to increased sensitivity to cisplatin and carboplatin. Similarly, patients with *BARD1* SNP rs2070096 were more likely to relapse in patients receiving a TCH-based treatment (docetaxel (T), carboplatin (C), and trastuzumab (H)).

*ERCC2 (cisplatin)*

8. Van Allen EM et al. Somatic ERCC2 mutations correlate with cisplatin sensitivity in muscle-invasive urothelial carcinoma. *Cancer Discov.* **4(10):**1140-53 (2014).

- This study reported that *ERCC2* was significantly mutated in cisplatin-sensitive cells (compared to resistant cells), and showed cell lines expressing wildtype *ERCC2* to be less sensitive to cisplatin compared to mutated *ERCC2 in vitro*.

9. Furuta T, Ueda T, Aune G, Sarasin A, Kraemer KH, Pommier Y. Transcription-coupled nucleotide excision repair as a determinant of cisplatin sensitivity of human cells. *Cancer Res.* **62(17)**:4899–4902 (2002).

- This study observes that TC-NER-deficient cells (such as *ERCC2* [XP-D]) are sensitive to cisplatin treatment, and that increased *ERCC2* [*XPD*] increases cisplatin resistance.

10. Aloyz R, Xu ZY, Bello V, Bergeron J, Han FY, Yan Y *et al*. Regulation of cisplatin resistance and homologous recombinational repair by the TFIIH subunit XPD. *Cancer Res.***62(19)**:5457–5462 (2002).

- Aloyz *et al.* report that the overexpression of *XPD* [*ERCC2*] in SK-MG-4 cells would increase the resistance of the cell to cisplatin without altering NER activity.

11. Li Q et al. ERCC2 Helicase Domain Mutations Confer Nucleotide Excision Repair Deficiency and Drive Cisplatin Sensitivity in Muscle-Invasive Bladder Cancer. *Clin Cancer Res.* 2018 Jul 6. [Epub ahead of print]

- This study introduces mutations into the helicase domain of *ERCC2*, and shows that these mutations can increase sensitivity of cells to cisplatin.

12. Qiang Li, Andrew Bell, Emmet Jordan, Sizhi Paul Gao, Jennifer Ma, Eugene J. Pietzak, Guido Dalbagni, Bernard H. Bochner, Jonathan E. Rosenberg, Dean F. Bajorin, David B. Solit, Nadeem Riaz, and Gopa Iyer. Effect of defective ERCC2 on cisplatin and ionizing radiation (IR) sensitivity in bladder cancer cells. *Journal of Clinical Oncology.* **35**:6_suppl, 333-333 (2017).

- Qiang *et al.* reported that cells with mutated *ERCC2* would have greater cisplatin sensitivity compared to parent cells without the mutation.

*POLD1 (Cisplatin)*

- *POLD1* is an error prone DNA polymerase, similar to other POL gene family members have been shown to sensitize cells to cisplatin. By MFA and SVM analysis, expression of *POLD1* contributes to the cisplatin response. Published studies citing this gene family include:

13. Albertella MR, Green CM, Lehmann AR, and O’Connor MJ. A role for polymerase eta in the cellular tolerance to cisplatin-induced damage. *Cancer Res.* **65(21):**9799 –9806 (2005).

- This report shows that XP-V cells without functional polymerase eta (*POLH*) are dramatically more sensitive to cisplatin compared to cells with functional polymerase eta.

14. Srivastava AK, Han C, Zhao R, et al. Enhanced expression of DNA polymerase eta contributes to cisplatin resistance of ovarian cancer stem cells. *Proceedings of the National Academy of Sciences of the United States of America*. **112(14)**:4411-4416 (2015).

- Srivastava *et al.* show that an increase in expression of *POLH* will increase cisplatin resistance in ovarian cancer stem cells.

15. Y. Li, X. Gao, J.Y. Wang. Comparison of two POLQ mutants reveals that a polymerase-inactive POLQ retains significant function in tolerance to etoposide and gamma-irradiation in mouse B cells. *Genes Cells.* **16(9):**973-983 (2011).

- In this report, the deletion of *Polq* in murine CH12B-lymphoma cells sensitized them to cisplatin.

*PRKCA (Cisplatin)*

16. Muscella A, Vetrugno C, Antonaci G, Cossa LG, Marsigliante S. PKC-δ/PKC-α activity balance regulates the lethal effects of cisplatin. *Biochem Pharmacol.* **98(1)**:29-40 (2015).

- This study reports that the inhibition of PKC- α (*PRKCA*) increased cytotoxicity of cisplatin, and suggested that PKC-α expression is involved in a pro-survival response to cisplatin treatment.

17. Spitaler M, Wiesenhofer B, Biedermann V, Seppi T, Zimmermann J, Grunicke H, Hofmann J. The involvement of protein kinase C isoenzymes alpha, epsilon and zeta in the sensitivity to antitumor treatment and apoptosis induction. *Anticancer Res.* **19(5B):**3969-76 (1999).

- Spitaler *et al.* report that the overexpression of PKC-α (*PRKCA*) in NIH3T3 cells would increase resistance of these cells to cisplatin.

18. Gabriel M et al. Role of the splicing factor SRSF4 in cisplatin-induced modifications of pre-mRNA splicing and apoptosis. *BMC Cancer.* **15:**227 (2015).

- It is observed in this manuscript that cisplatin treatment will decrease the overall expression of *PRKCA*.

19. Kong LR et al. MEK Inhibition Overcomes Cisplatin Resistance Conferred by SOS/MAPK Pathway Activation in Squamous Cell Carcinoma. *Mol Cancer Ther.* **14(7):**1750-60 (2015).

- This article shows that *PRKCA* is down-regulated in cisplatin-sensitive lung SCC cell lines when treated with cisplatin.

*PRKCB (Cisplatin)*

20. Li N, Zhang W. Protein kinase C β inhibits autophagy and sensitizes cervical cancer Hela cells to cisplatin. *Biosci Rep*. **37**(2) (2017).

- This study reports that the inhibition of PKC β (*PRKCB*) in cervical cancer Hela cells would increase cell resistance to cisplatin treatment, while overexpression of the gene sensitized the cells to cisplatin.

*AKT1 (Cisplatin and Carboplatin)*

21. Lee CS, Kim YJ, Jang ER, Myung SC, Kim W. Akt inhibitor enhances apoptotic effect of carboplatin on human epithelial ovarian carcinoma cell lines. *Eur J Pharmacol.* **632(1-3):**7-13 (2010).

- It is reported in this study that the apoptosis of epithelial ovarian cancer cell lines OVCAR-3 and SK-OV-3 by carboplatin is enhanced by the inclusion of an AKT inhibitor.

22. Carlson et al. Abstract 5451: Testing a combination of AKT inhibitor (AZD5363) with PARP inhibitor olaparib plus carboplatin in TNBC. *Cancer Res.* (78) (13 Supplement) 5451 (2018).

- This abstract describes experiments where treatment with the AKT inhibitor AZD5363 in PTEN-null SUM149 cells (with *BRCA1* mutation) responded synergistically with carboplatin.

23. Hovelmann S, Beckers TL, Schmidt M. Molecular alterations in apoptotic pathways after PKB/Akt-mediated chemoresistance in NCI H460 cells. *Br J Cancer.* **90 (12):**2370–7 (2004).

- Hovelmann *et al.* reports that NCI H460 with increased expression of Akt1 led to a 10-fold decrease in sensitivity to cisplatin compared to control NCI H460 cells.

24. Hahne JC, Honig A, Meyer SR, Gambaryan S, Walter U, Wischhusen J, Häussler SF, Segerer SE, Fujita N, Dietl J, Engel JB. Downregulation of AKT reverses platinum resistance of human ovarian cancers in vitro. *Oncol Rep.* **28(6):**2023-8 (2012).

- It is reported by Hahne *et al.* that the downregulation of *AKT* (*AKT1*) in the ovarian cancer cell line A2780cis reversed platinum resistance (only cisplatin was tested).

*ERCC1 (Carboplatin)*

25. M. Dabholkar, F. Bostick-Bruton, C. Weber, V.A. Bohr, C. Egwuagu, E. Reed. ERCC1 and ERCC2 expression in malignant tissues from ovarian cancer patients. *J Natl Cancer Inst.* **84(19):**1512-1517 (1992).

- This paper reports that patients resistant to a cis- or carbo-platin treatment protocol had 2.6 fold higher expression of ERCC1 compared to responding patients.

26. Du P, Wang Y, Chen L, Gan Y, Wu Q. High ERCC1 expression is associated with platinum-resistance, but not survival in patients with epithelial ovarian cancer. *Oncol Lett.* **12(2)**:857-862 (2016).

- In this article, there was a strong association of ERCC1 expression and platinum-resistant patients (treatment regiment included either cisplatin or carboplatin).

27. S. Kang, W. Ju, J.W. Kim, et al. Association between excision repair cross-complementation group 1 polymorphism and clinical outcome of platinum-based chemotherapy in patients with epithelial ovarian cancer. *Exp Mol Med.* **38(3):**320-324 (2006).

- Kang et al. report that an ERCC1 polymorphism (Asn118Asn) was predictive of sensitivity to platinum-resistance (carboplatin, specifically) in ovarian cancer patients.

*MTHFR (Carboplatin)*

28. Cui LH, Yu Z, Zhang TT, Shin MH, Kim HN, Choi JS. Influence of polymorphisms in MTHFR 677 C→T, TYMS 3R→2R and MTR 2756 A→G on NSCLC risk and response to platinum-based chemotherapy in advanced NSCLC. *Pharmacogenomics.* **12(6):**797-808 (2011).

- This study reports a better response to cisplatin or carboplatin-based chemotherapy for NSCLC patients with the TT allele of the *MTHFR* polymorphism 677 C→T.

29. Smit EF, Burgers SA, Biesma B, Smit HJ, Eppinga P, Dingemans AM, Joerger M, Schellens JH, Vincent A, van Zandwijk N, Groen HJ. Randomized phase II and pharmacogenetic study of pemetrexed compared with pemetrexed plus carboplatin in pretreated patients with advanced non-small-cell lung cancer. *J Clin Oncol.* **27(12):**2038-45 (2009).

- This report also found that NSCLC patients homozygous for *MTHFR* C677T had improved survival to chemotherapy (pemetrexed and/or carboplatin). They also report a lower survival for patients homozygous with *MTHFR* 1298C (not statistically significant; p = 0.06).

*MSH2 (Cisplatin and Oxaliplatin)*

30. Alex, A. K. et al. Response to Chemotherapy and Prognosis in Metastatic Colorectal Cancer With DNA Deficient Mismatch Repair. *Clin. Colorectal Cancer.* **16(3):**228-239 (2016).

- This study reports that a deficiency in DNA mismatch repair genes, such as *MSH2*, is predictive for oxaliplatin resistance.

31. Raymond, E., Faivre, S., Chaney, S., Woynarowski, J. & Cvitkovic, E. Cellular and Molecular Pharmacology of Oxaliplatin. *Mol. Cancer Ther.* **1(3):**227-35 (2002).

- Raymond *et al.* states that *MLH1*, *MSH2* and *MSH6*-deficient cells are more susceptible to oxaliplatin, despite MMR-deficiency being associated with cisplatin resistance.

*IGF1 (Oxaliplatin)*

32. E. Volkova, B.A. Robinson, J. Willis, M.J. Currie, G.U. Dachs. Marginal effects of glucose, insulin and insulin-like growth factor on chemotherapy response in endothelial and colorectal cancer cells. *Oncol Lett.* **7(2):**311-320 (2014).

- In this report, the induction of IGF-1 (*IGF1*) combined with low dose chemotherapy (including oxaliplatin) increased WiDr colorectal cancer cell viability.

*IGF1R (Oxaliplatin)*

33. Dallas NA, Xia L, Fan F, Gray MJ, Gaur P, van Buren G 2nd, Samuel S, Kim MP, Lim SJ, Ellis LM. Chemoresistant colorectal cancer cells, the cancer stem cell phenotype, and increased sensitivity to insulin-like growth factor-I receptor inhibition. *Cancer Res.* **69(5):**1951-7 (2009).

- It is reported that T29 colorectal cancer cells that were found to be resistance to 5-FU and oxaliplatin showed increased expression and activation of IGF-1R (*IGF1R*).

*NOTCH1 (Oxaliplatin)*

34. Meng RD , et al. γ-Secretase inhibitors abrogate oxaliplatin-induced activation of the Notch-1 signaling pathway in colon cancer cells resulting in enhanced chemosensitivity. *Cancer Res.* **69(2):**573-582 (2009).

- This article reports that the inhibition of Notch-1 (*NOTCH1*) with siRNA chemosensitized colon cancer cells to oxaliplatin.

35. Huang R, et al. Colorectal cancer stem cell and chemoresistant colorectal cancer cell phenotypes and increased sensitivity to Notch pathway inhibitor. *Mol Med Rep.* **12(2):**2417-24 (2015).

- In Huang *et al.*, Notch1 levels were shown to be higher in oxaliplatin resistant HCT116 cell lines compared to parental cells. Furthermore, the introduction of a Notch pathway inhibitor (DAPT) was shown to increase sensitivity to oxaliplatin.

36. Kukcinaviciute E et al. Significance of Notch and Wnt signaling for chemoresistance of colorectal cancer cells HCT116. *J Cell Biochem.* **119(7):**5913-5920 (2018).

- This study reports that the Notch inhibitor RO4929097 improves survival of oxaliplatin-treated HCT116 cells.

**Supplementary References, Section B.** Initial Peer-Reviewed Literature

1. Albertella, M.R. *et al.* A role for polymerase eta in the cellular tolerance to cisplatin-induced damage. *Cancer Res.* **65(21)**,9799-806 (2005).

2. Alex, A.K. *et al*. Response to Chemotherapy and Prognosis in Metastatic Colorectal Cancer With DNA Deficient Mismatch Repair. *Clin Colorectal Cancer.* **16(3)**,228-239 (2017).

3. Baba, H. *et al*. Changes in expression levels of ERCC1, DPYD, and VEGFA mRNA after first-line chemotherapy of metastatic colorectal cancer: results of a multicenter study. *Oncotarget.* **6(32)**, 34004-13 (2015).

4. Basu, A. and Krishnamurthy, S. Cellular responses to Cisplatin-induced DNA damage. *J Nucleic Acids.* pii: 201367 (2010).

5. Bhatt, M. *et al*. Drug-dependent functionalization of wild-type and mutant p53 in cisplatin-resistant human ovarian tumor cells. *Oncotarget.* **8(7)**, 10905-10918 (2017).

6. Bommer, U.A. *et al*. Translationally controlled tumour protein TCTP is induced early in human colorectal tumours and contributes to the resistance of HCT116 colon cancer cells to 5-FU and oxaliplatin. *Cell Commun Signal.* **15(1),**9 (2017). .

7. Borst, P. *et al*. How do real tumors become resistant to cisplatin? *Cell Cycle.* **7,**1353–9 (2008).

8. Burger, H. *et al*. Differential transport of platinum compounds by the human organic cation transporter hOCT2 (hSLC22A2). *Br J Pharmacol.* **159(4),**898-908 (2010).

9. Cadoná, F.C. *et al*. Guaraná a Caffeine-Rich Food Increases Oxaliplatin Sensitivity of Colorectal HT-29 Cells by Apoptosis Pathway Modulation. *Anticancer Agents Med Chem.* **16(8),** 1055-1065 (2016)

10. Ceckova, M. *et al*. Effect of ABCG2 on cytotoxicity of platinum drugs: interference of EGFP. *Toxicol In Vitro.* **22(8),** 1846-52 (2008).

11. Chen, C.C. *et al*. Gene expression profiling for analysis acquired oxaliplatin resistant factors in human gastric carcinoma TSGH-S3 cells: the role of IL-6 signaling and Nrf2/AKR1C axis identification. *Biochem Pharmacol.* **86(7)**, 872-87 (2013).

12. Chian, S. *et al*. Luteolin sensitizes two oxaliplatin-resistant colorectal cancer cell lines to chemotherapeutic drugs via inhibition of the Nrf2 pathway. *Asian Pac J Cancer Prev.* **15(6)**, 2911-6 (2014).

13. Cho, Y.B. *et al*. Relationship between TYMS and ERCC1 mRNA expression and in vitro chemosensitivity in colorectal cancer. *Anticancer Res.* **31(11),** 3843-9 (2011).

14. Choi, W. *et al*. Identification of Distinct Basal and Luminal Subtypes of Muscle-Invasive Bladder Cancer with Different Sensitivities to Frontline Chemotherapy. *Cancer Cell.* **25,** 152–65 (2014).

15. Cinci, L. *et al.* Effects of Hypericum perforatum extract on oxaliplatin-induced neurotoxicity: in vitro evaluations. *Z Naturforsch C.* **72(5-6),** 219-226 (2017). doi: 10.1515/znc-2016-0194.

16. Custodio, A. *et al*. Pharmacogenetic predictors of severe peripheral neuropathy in colon cancer patients treated with oxaliplatin-based adjuvant chemotherapy: a GEMCAD group study. *Ann Oncol.* **25(2),** 398-403 (2014).

17. Custodio, A. *et al*. Pharmacogenetic predictors of outcome in patients with stage II and III colon cancer treated with oxaliplatin and fluoropyrimidine-based adjuvant chemotherapy. *Mol Cancer Ther.* **13(9)**, 2226-37 (2014).

18. Dabkeviciene, D. *et al*. The role of interleukin-8 (CXCL8) and CXCR2 in acquired chemoresistance of human colorectal carcinoma cells HCT116. *Med Oncol.* **32(12),** 258 (2015).

19. Galluzzi, L. *et al.* Molecular mechanisms of cisplatin resistance. *Oncogene.* **31,** 1869–83 (2012).

20. Geng, R. *et al*. Oxidative stress-related genetic polymorphisms are associated with the prognosis of metastatic gastric cancer patients treated with epirubicin, oxaliplatin and 5-fluorouracil combination chemotherapy. *PLoS One.* **9(12),** e116027 (2014).

21. Goekkurt, E. *et al*. Pharmacogenetic analyses of a phase III trial in metastatic gastroesophageal adenocarcinoma with fluorouracil and leucovorin plus either oxaliplatin or cisplatin: a study of the arbeitsgemeinschaft internistische onkologie. *J Clin Oncol.* **27(17),** 2863-73 (2009).

22. Hall, J.C. *et al*. Novel patient-derived xenograft mouse model for pancreatic acinar cell carcinoma demonstrates single agent activity of oxaliplatin. *J Transl Med.* **14(1)**, 129 (2016)

23. Hariani, G.D. *et al*. Application of next generation sequencing to CEPH cell lines to discover variants associated with FDA approved chemotherapeutics. *BMC Res Notes.* **7,** 360 (2014).

24. Inanç, M. *et al*. Prognostic value of tumor growth factor levels during chemotherapy in patients with metastatic colorectal cancer. *Med Oncol.* **29(5)**, 3119-24 (2012).

25. Jiang, S.P. *et al*. Celecoxib reverts oxaliplatin-induced neuropathic pain through inhibiting PI3K/Akt2 pathway in the mouse dorsal root ganglion. *Exp Neurol.* **275** Pt 1:11-6 (2016).

26. Jin, Y.Y. *et al*. Involvement of microRNA-141-3p in 5-fluorouracil and oxaliplatin chemo-resistance in esophageal cancer cells via regulation of PTEN. *Mol Cell Biochem.* **422(1-2), 1**61-170 (2016).

27. Johnson, B. *et al*. Next generation sequencing identifies 'interactome' signatures in relapsed and refractory metastatic colorectal cancer. *J Gastrointest Oncol.* **8(1),** 20-31 (2017).

28. Johnson, B. *et al*. Molecular profiling of a case of advanced pancreatic cancer identifies an active and tolerable combination of targeted therapy with backbone chemotherapy. *J Gastrointest Oncol.* **(2),** E6-E12 (2016).

29. Kap, E.J. *et al*. Genetic variants in the glutathione S-transferase genes and survival in colorectal cancer patients after chemotherapy and differences according to treatment with oxaliplatin. *Pharmacogenet Genomics.* **24(7),** 340-7 (2014).

30. Kim, S.Y. *et al*. S-1 plus irinotecan and oxaliplatin for the first-line treatment of patients with metastatic colorectal cancer: a prospective phase II study and pharmacogenetic analysis. *Br J Cancer.* **109(6),** 1420-7 (2013).

31. Kjersem, J.B. *et al*. FCGR2A and FCGR3A polymorphisms and clinical outcome in metastatic colorectal cancer patients treated with first-line 5-fluorouracil/folinic acid and oxaliplatin +/- cetuximab. *BMC Cancer*. **14,** 340 (2014).

32. Kjersem, J.B. *et al*. AGXT and ERCC2 polymorphisms are associated with clinical outcome in metastatic colorectal cancer patients treated with 5-FU/oxaliplatin. *Pharmacogenomics J.* **16(3),** 272-9 (2016).

33. Li, X.Z. *et al*. Oxaliplatin-rapamycin combination was superior to mono-drug in treatment of hepatocellular carcinoma both in vitro and in vivo. *Neoplasma.* **63(6),** 880-887 (2016).

34. Li, Y. *et al*. Predictive value of CHFR and MLH1 methylation in human gastric cancer. *Gastric Cancer.* **18(2),** 280-7 (2015).

35. Li, Z. *et al*. ABCC2-24C > T polymorphism is associated with the response to platinum/5-Fu-based neoadjuvant chemotherapy and better clinical outcomes in advanced gastric cancer patients. *Oncotarget.* **7(34)**, 55449-55457 (2016).

36. Liu, X. *et al*. Effects of IGF2BP2, KCNQ1 and GCKR polymorphisms on clinical outcome in metastatic gastric cancer treated with EOF regimen. *Pharmacogenomics.* **16(9),** 959-70 (2015).

37. Liu, Y. *et al*. Prediction of genetic polymorphisms of DNA repair genes XRCC1 and XRCC3 in the survival of colorectal cancer receiving chemotherapy in the Chinese population. *Hepatogastroenterology.* **59(116),** 977-80 (2012).

38. Lu, M. *et al*. Activation of the mTOR Pathway by Oxaliplatin in the Treatment of Colorectal Cancer Liver Metastasis. *PLoS One.* **12(1),** e0169439 (2017).

39. Masek, V. *et al*. Interaction of antitumor platinum complexes with human liver microsomal cytochromes P450. *Anticancer Drugs*. **20(5),** 305-11 (2009).

40. Masek, V. *et al*. Interaction of selected platinum(II) complexes containing roscovitine-based CDK inhibitors as ligands with human liver microsomal cytochrome P450. *Biomed Pap Med Fac Univ Palacky Olomouc Czech Repub.* **159(3),** 382-7 (2015).

41. Mezquita, B. *et al*. LoVo colon cancer cells resistant to oxaliplatin overexpress c-MET and VEGFR-1 and respond to VEGF with dephosphorylation of c-MET. *Mol Carcinog.* **55(5),** 411-9 (2016).

42. Mo, D. *et al.* Human Helicase RECQL4 Drives Cisplatin Resistance in Gastric Cancer by Activating an AKT-YB1-MDR1 Signaling Pathway. *Cancer Res.***76,** 3057–66 (2016).

43. Montazami, N. *et al*. siRNA-mediated silencing of MDR1 reverses the resistance to oxaliplatin in SW480/OxR colon cancer cells. *Cell Mol Biol (Noisy-le-grand).* **61(2),** 98-103 (2015).

44. Monzo, M. *et al*. Single nucleotide polymorphisms in nucleotide excision repair genes XPA, XPD, XPG and ERCC1 in advanced colorectal cancer patients treated with first-line oxaliplatin/fluoropyrimidine. *Oncology*. **72(5-6),** 364-70 (2007).

45. Ning, Y. *et al*. The CXCR2 antagonist, SCH-527123, shows antitumor activity and sensitizes cells to oxaliplatin in preclinical colon cancer models. *Mol Cancer Ther.* **11(6),** 1353-64 (2012).

46. Oguri, T. *et al*. Genetic polymorphisms associated with oxaliplatin-induced peripheral neurotoxicity in Japanese patients with colorectal cancer. *Int J Clin Pharmacol Ther.* **51(6),** 475-81 (2013). doi: 10.5414/CP201851.

47. Paré-Brunet, L. *et al*. Genetic variations in the VEGF pathway as prognostic factors in metastatic colorectal cancer patients treated with oxaliplatin-based chemotherapy. *Pharmacogenomics J.* **15(5),** 397-404 (2015). doi: 10.1038/tpj.2015.1. Epub 2015 Feb 24.

48. Park, J.H. *et al*. MGMT -535G>T polymorphism is associated with prognosis for patients with metastatic colorectal cancer treated with oxaliplatin-based chemotherapy. *J Cancer Res Clin Oncol.* **136(8),** 1135-42 (2010).

49. Pires, I.M. *et al*. Oxaliplatin responses in colorectal cancer cells are modulated by CHK2 kinase inhibitors. *Br J Pharmacol.* **159(6),** 1326-38 (2010).

50. Planutis, A.K. *et al*. SW480 colorectal cancer cells that naturally express Lgr5 are more sensitive to the most common chemotherapeutic agents than Lgr5-negative SW480 cells. *Anticancer Drugs.* **26(9),** 942-7 (2015).

51. Plasencia, C. *et al*. Expression analysis of genes involved in oxaliplatin response and development of oxaliplatin-resistant HT29 colon cancer cells. *Int J Oncol.* **29(1),** 225-35 (2006).

52. Poisson, L.M. *et al*. A metabolomic approach to identifying platinum resistance in ovarian cancer. *J Ovarian Res [Internet]*. [cited 2015 Oct 27];8 (2015). Available from: http://ovarianresearch.biomedcentral.com/articles/10.1186/s13048-015-0140-8

53. Song, I.S. *et al*. Role of human copper transporter Ctr1 in the transport of platinum-based antitumor agents in cisplatin-sensitive and cisplatin-resistant cells. *Mol Cancer Ther.* **3(12),** 1543-9 (2004).

54. Song, W. and Ma, H. The expression of ERCC1 and BRCA1 predicts prognosis of platinum-based chemotherapy in urothelial cancer. *Onco Targets Ther.* **9,** 3465-71 (2016).

55. Alcindor, T. and Beauger, N. Oxaliplatin: a review in the era of molecularly targeted therapy. *Curr Oncol.* **18(1),** 18-25 (2011).

56. Thomaidis, T. *et al*. VEGFR-3 and CXCR4 as predictive markers for treatment with fluorouracil, leucovorin plus either oxaliplatin or cisplatin in patients with advanced esophagogastric cancer: a comparative study of the Arbeitsgemeinschaft Internistische Onkologie (AIO). *BMC Cancer.* **14,** 476 (2014).

57. Turkington, R.C. *et al*. Fibroblast growth factor receptor 4 (FGFR4): a targetable regulator of drug resistance in colorectal cancer. *Cell Death Dis.* **5,** e1046 (2014).

58. Varma, R.R. *et al*. Gene expression profiling of a clonal isolate of oxaliplatin-resistant ovarian carcinoma cell line A2780/C10. *Oncol Rep.* **14(4),** 925-32 (2005).

59. von Stechow, L. *et al*. Identification of Cisplatin-Regulated Metabolic Pathways in Pluripotent Stem Cells. Santos J, editor. *PLoS ONE.***8,** e76476 (2013).

60. Vondálová Blanářová, O. *et al*. Higher anti-tumour efficacy of platinum(IV) complex LA-12 is associated with its ability to bypass M-phase entry block induced in oxaliplatin-treated human colon cancer cells. *Cell Prolif.* **46(6),** 665-76 (2013).

61. Wagner, J.M. and Karnitz, L.M. Cisplatin-induced DNA damage activates replication checkpoint signaling components that differentially affect tumor cell survival. *Mol Pharmacol.* **76(1),** 208-14 (2009).

62. Wei, Y. *et al*. Synergistic cytotoxicity from combination of imatinib and platinum-based anticancer drugs specifically in Bcr-Abl positive leukemia cells. *J Pharmacol Sci.* **129(4),** 210-5 (2015).

63. Wernyj, R. and Morin, P. Molecular mechanisms of platinum resistance: still searching for the Achilles? heel. *Drug Resist Updat.* **7,** 227–32 (2004).

64. Won, H.H. *et al*. Polymorphic markers associated with severe oxaliplatin-induced, chronic peripheral neuropathy in colon cancer patients. *Cancer.* **118(11),** 2828-36 (2012).

65. Xin-Xiang Li, *et. al*. RNA-seq identifies determinants of oxaliplatin sensitivity in colorectal cancer cell lines. *Int J Clin Exp Pathol.* **7(7),** 3763-3770 (2014).

66. Xu, P. *et al*. Upregulated HOXC8 Expression Is Associated with Poor Prognosis and Oxaliplatin Resistance in Hepatocellular Carcinoma. *Dig Dis Sci.* **60(11),** 3351-63 (2015).

67. Yang, C. *et al*. Effects of PEG-liposomal oxaliplatin on apoptosis, and expression of Cyclin A and Cyclin D1 in colorectal cancer cells. *Oncol Rep.* **28(3)**, 1006-12 (2012).

68. Ye, Y. *et al*. MDM2 is a useful prognostic biomarker for resectable gastric cancer. *Cancer Sci.* **104(5),** 590-8 (2013).

69. Yokoo, S. *et al*. Significance of organic cation transporter 3 (SLC22A3) expression for the cytotoxic effect of oxaliplatin in colorectal cancer. *Drug Metab Dispos.* **36(11),** 2299-306 (2008).

70. Zeng, Z.L. *et al*. Overexpression of the circadian clock gene Bmal1 increases sensitivity to oxaliplatin in colorectal cancer. *Clin Cancer Res.* **20(4),** 1042-52 (2014).

71. Zhang, L. *et al*. Polymorphisms of ERCC1 and XRCC1 predict the overall survival of advanced gastric cancer patients receiving oxaliplatin-based chemotherapy. *Int J Clin Exp Med.* **8(10),** 18375-82 (2015).

72. Zhang, S. *et al*. Organic cation transporters are determinants of oxaliplatin cytotoxicity. *Cancer Res.* **66(17),** 8847-57 (2006).

73. Zhao, Q. *et al*. Enhancement of Drug Sensitivity by Knockdown of HIF-1α in Gastric Carcinoma Cells. *Oncol Res.* **23(3),** 129-36 (2016).
